# Parkinson’s disease and gut microbiota: from clinical to mechanistic and therapeutic studies

**DOI:** 10.1186/s40035-023-00392-8

**Published:** 2023-12-15

**Authors:** Xuxiang Zhang, Beisha Tang, Jifeng Guo

**Affiliations:** 1 Department of Neurology, Xiangya Hospital, Central South University, Changsha, 410008 China; 2 Key Laboratory of Hunan Province in Neurodegenerative Disorders, Central South University, Changsha, 410008 China; 3 Hunan International Scientific and Technological Cooperation Base of Neurodegenerative and Neurogenetic Diseases, Changsha, 410008 China; 4 Center for Medical Genetics and Hunan Key Laboratory of Medical Genetics, School of Life Sciences, Central South University, Changsha, 410008 China; 5 Engineering Research Center of Hunan Province in Cognitive Impairment Disorders, Central South University, Changsha, 410008 China; 6 National Clinical Research Center for Geriatric Disorders, Xiangya Hospital, Central South University, Changsha, 410008 China

**Keywords:** Gut microbiota, Microbiota–gut–brain axis, Mechanisms, Alpha-synuclein, Microbial therapy

## Abstract

Parkinson’s disease (PD) is one of the most prevalent neurodegenerative diseases. The typical symptomatology of PD includes motor symptoms; however, a range of nonmotor symptoms, such as intestinal issues, usually occur before the motor symptoms. Various microorganisms inhabiting the gastrointestinal tract can profoundly influence the physiopathology of the central nervous system through neurological, endocrine, and immune system pathways involved in the microbiota–gut–brain axis. In addition, extensive evidence suggests that the gut microbiota is strongly associated with PD. This review summarizes the latest findings on microbial changes in PD and their clinical relevance, describes the underlying mechanisms through which intestinal bacteria may mediate PD, and discusses the correlations between gut microbes and anti-PD drugs. In addition, this review outlines the status of research on microbial therapies for PD and the future directions of PD–gut microbiota research.

**Supplementary Information:**

The online version contains supplementary material available at 10.1186/s40035-023-00392-8.

## Introduction

Parkinson’s disease (PD) is a relatively common neurodegenerative disease in the elderly population. The exact factor that triggers PD is still unclear; however, its development is driven by a mixture of genetic and environmental variables. Given that fewer than 10% of cases are attributable to certain genetic factors, determining the environmental risk factors for PD is important [1, 2].

The intestine serves as a gateway to the environment, through which environmental variables can influence the pathogenesis and evolution of PD. Although the gut has immune and physical barrier functions [3, 4] that can protect it from environmental damage, these functions deteriorate with age [5, 6], resulting in increased opportunities for the body to be exposed to potentially harmful environmental elements [7]. Braak et al. [8] have proposed an “ascending anatomical theory”, which implies that PD evolves from the gut toward the brain. Several studies have accumulated considerable evidence both for and against this theory (Table 1).

**Table 1:** Evidence for and against the gut origin of PD

Evidence for	Evidence against
*Epidemiological studies*	
Gastrointestinal symptoms usually precede the motor symptoms of PD [18].	CNS and PNS are simultaneously involved in PD, with peripheral symptoms appearing first owing to poorer compensatory mechanisms [19].
IBD increases the incidence of PD [20–24]. Effective treatment can reduce the risk of PD [23, 25].	A retrospective study did not confirm that IBD increases the risk for PD [26]. The results of a Mendelian randomization study did not support that treating IBD could prevent PD [27].
Vagotomy and appendectomy can lower the risk of PD [28, 29].	A long-term follow-up study did not confirm that vagotomy reduces the risk of PD [30]. In most studies, appendectomy is not correlated with PD; rather it even slightly increases the risk of PD in some studies [31–33].
*Neuropathological studies*	
Pathological changes in PD may first occur in the ENS [34].	Results of several clinicopathological studies do not support the peripheral origin of PD. The studies showed that α-syn histopathology of the PNS rarely precedes the CNS [35–37].
Increased intestinal permeability and decreased level of the tight junction protein occludin in PD [38–40].	
*Clinical studies*	
Intestinal flora dysbiosis can occur in the prodromal phase of PD [41].	
Gut microbes are associated with motor and nonmotor PD phenotypes [42].	
Microbial therapy can improve the clinical manifestations of PD [43].	
*Animal studies*	
Changes in intestinal flora produce abnormal metabolites and structural proteins, which may trigger α-syn accumulation [44, 45].	The origin of PD may be multifocal [19].
α-Syn originates in the gut and spreads to the CNS through a transsynaptic intercellular approach [46].	PD pathologies, such as α-syn overexpression, can also propagate from the CNS to the intestine [47–51].
Fecal microbiome transplantation can exacerbate or improve PD-like symptoms in animal models [45].	

PD: Parkinson’s disease, CNS: central nervous systems, PNS: peripheral nervous systems, IBD: inflammatory bowel disease, ENS: enteric nervous system, α-syn: alpha-synuclein

Borghammer and colleagues [9–14] recently proposed the body-first and brain-first hypotheses of PD, in which autonomic damage and dopaminergic dysfunction are proposed to appear in different chronological sequences. In the body-first subtype, the pathology originates in the gut or the peripheral autonomic nervous system. It is accompanied by more common prodromal autonomic symptoms, such as rapid eye movement sleep behavior disorder. In contrast, the brain-first subtype has pathology that initially appears in the midbrain or olfactory bulb, has a shorter prodromal period, and has fewer nonmotor symptoms before diagnosis. A large body of evidence has accumulated in recent years regarding the peripheral origin of PD. Gastrointestinal dysfunction has been detected in patients prior to the diagnosis of PD, and intestinal neuronal innervation may have been affected in the early stage of PD [15]. Imaging data have shown significant dysfunction of the parasympathetic and sympathetic nervous systems in patients with idiopathic rapid eye movement sleep behavior disorder, similar to patients with PD [16]. Pathological investigations have also indicated that α-syn misfolding and aggregation in idiopathic rapid eye movement sleep behavior disorder occurs in the peripheral nervous system and then travels rostrally to the brainstem [15, 17].

## Gastrointestinal dysfunction and PD

Gastrointestinal dysfunction is the main early nonmotor symptom of PD. Up to 80% of patients with PD may experience gastrointestinal problems [52, 53]. Constipation is particularly common in PD [54] and occurs years or even decades before the motor symptoms of PD [18, 55]. Some epidemiological studies have demonstrated that constipation increases the risk of PD to some extent [56]. In addition, constipation indicates a worse disease course in PD; for example, constipation in the early stages of PD increases the risk of dementia [57]. The considerable evidence from previous studies has led the International Movement Disorders Society (MDS) to include constipation as a clinical biomarker of PD in its diagnostic criteria for the prodromal phase of PD [58].

### PD may originate in the intestine

Alpha-synuclein (α-syn) inclusions were first discovered in the 1980s in the enteric tissues of patients with PD, leading researchers to speculate that the gastrointestinal system may be implicated in the pathogenesis of PD [59–61]. Subsequently, Braak et al. [8, 62] proposed that pathogenic α-syn inclusions may begin in the gastrointestinal system [63, 64]. Epidemiological studies have shown that vagotomy and appendectomy may reduce the risk of developing PD [28, 29]. Results of some animal studies also support this hypothesis. In a rat model, Kim et al. [65] demonstrated that truncal vagotomy prevents transneuronal transmission of α-syn from the gut to the central nervous systems (CNS). In addition, it has been proven that α-syn spreads to the CNS and higher cortical regions through transsynaptic cell-to-cell transmission [46]. However, reliable evidence that supports the current view on the gut origin of α-syn is still lacking. In addition, some studies have shown contradictory results. For example, some studies proposed that α-syn transmission along the gut–brain axis may be bidirectional [48, 66, 67]. Furthermore, evidence from autopsies does not support the peripheral origin of PD. Notably, in a previous study, analysis of whole-body autopsy data from the Arizona Study of Aging and Neurodegenerative Disorders revealed few autopsy cases of peripheral α-syn pathology without CNS involvement [36]. However, this study does not serve as compelling evidence against the body-first hypothesis of PD, as clarifying the α-syn pathology of the gut is extremely challenging because of factors such as the scope, the time, and the methodology of the assay, and the high probability of test misses [13, 68].

## Gut microbes and the microbiota–gut–brain axis

Gut microbes help regulate gastrointestinal and immune functions. They also affect the digestion and metabolism of several foods, nutrients, metabolites, and drugs [69–74]. The balance of gut microbes and its impact on human health have received great attention. Disruption of the gut microbiota balance has been linked to many human disorders, including gastrointestinal, neurological, metabolic, respiratory, and cardiovascular diseases [75]. Gut microbes are the pivotal hub of the gut–brain link and have been called the body’s second brain [76]. The term “microbiota–gut–brain” was coined to characterize this complex mechanism [77].

It is well known that PD affects not only the CNS but also the digestive system and the enteric nervous system (ENS). Pathologic changes in the ENS occur early in PD, even before pathologic changes in the CNS [78]. Research conducted on PD patients and animal models indicates the presence of neuronal and glial injury within the ENS [79]. Several research groups have reported Lewy-type pathology in biopsied enteric neurons from PD patients. Atrophic degeneration of neurons in the myenteric plexus and submucosal plexus with colocalized α-syn deposits has been reported in PD patients [80]. Constipation symptoms in patients may result from the dysfunction of vasoactive intestinal peptide secretomotor neurons in the submucosal plexus [81]. In addition, glial markers such as glial fibrillary acidic protein (GFAP) and Sox-10 are increased and associated with pro-inflammatory cytokines in the gastrointestinal tract of PD patients [82]. Animal studies have found that aged rats exhibit neuronal loss and changes of the ENS neurochemical phenotypes, accompanied by dystrophic enteric neurons which contain α-syn aggregates, and that pathologic α-syn can, in turn, affect the cytoskeleton of ENS neurons [78, 83]. In A30P transgenic mice overexpressing α-syn, the ENS is more sensitive to A30P α-syn than the CNS, and similarly, mice overexpressing wild-type human α-syn under the Thy1 promoter show alterations in the colonic myenteric ganglion several months prior to striatal dopamine loss [83, 84].

The gut**–**brain axis is involved in PD; thus, gut-related dysbiosis and alterations in microbial-derived components are risk factors and important determinants of PD [85]. Studying the mechanism by which the microbiota–gut–brain axis affects the neurological system can help clarify the etiology of PD.

## Gut microbiota in PD

### Dysbiosis in PD

Since the first gut dysbiosis-PD study [86] published in 2015, dozens of human case–control studies have been published to date (Table 2 and Additional file 1: Table S1). The sample sizes of these studies varied from tens to hundreds, although the inclusion and exclusion criteria may differ. For example, some studies included only drug-naïve PD or early PD, while some included only late-onset PD, or included only male patients to exclude the influence of gender. Consistently, however, most of these studies excluded subjects with concomitant intestinal diseases and recent use of antibiotics, as these factors significantly interfere with the detection of gut microbes. Similarly, some studies have matched the PD and the control groups regarding diet, medications, environmental factors, and other factors that may influence the gut microbiota. Further, a considerable number of studies have included spouses as the control group to eliminate the interference of confounding factors as much as possible.

**Table 2:** Microbiome alterations in clinical cohorts of PD

Ref.	Sample size	Control factors	Method	Microbiota alterations	Alpha diversity	Beta diversity
[86]	PD: 72 non-PD: 72	Onset age > 50 years; age- and sex-matched; no endocrine diseases	16S rRNA V1-3	Family increased: Lactobacillaceae, Verrucomicrobiaceae, Bradyrhizobiaceae, Clostridiales Incertae Sedis IV, Ruminococcaceae; family decreased: Prevotellaceae	nd	sd
[87]	PD: 31 non-PD: 28	Early, L-DOPA-naïve PD; only male; age-matched; diet and smoking habits considered	shotgun metagenomic	Family/genus increased: Verrucomicrobiaceae (genus *Akkermansia*); family/genus decreased: Prevotellaceae (genus *Prevotella*), Erysipelotrichaceae (genus *Eubacterium*); species increased: *Akkermansia muciniphila, Alistipes shahii;* species decreased: *Prevotella copri, Eubacterium bioforme, Clostridium saccharolyticum*	nd	sd
[88]	PD: 24 healthy: 14	Age-, sex-, BMI-matched; no diabetes, infectious diseases or special diets	16S rRNA V3-5	Family increased: Enterobacteriaceae, Veillonellaceae, Erysipelotrichaceae, Coriobacteriaceae, Streptococcaceae, Moraxellaceae, Enterococcaceae; genus increased: *Acidaminococcus, Acinetobacter, Enterococcus, Escherichia-Shigella, Megamonas, Megasphaera, Proteus, Streptococcus;* genus decreased: *Blautia, Faecalibacterium, Ruminococcus*	nd	sd
[89]	PD: 76 RBD: 21 healthy: 78	Comorbidities and comedication were documented	16S/18S rRNA V4	Family increased: Verrucomicrobiaceae; genus increased: *Akkermansia*	nd	sd
[90]	PD: 45 healthy: 45	Spouses as control; no serious chronic illnesses (e.g., diabetes); IBS excluded	16S rRNA V3-4	Genus increased: *Clostridium IV, Holdemania, Clostridium XVIII, Butyricicoccus, Anaerotruncus, Aquabacterium, Sphingomonas*	sd ( >)	sd
[91]	PD: 64 Control: 64	Age- and sex-matched; dietary habits and medications assessed	16S rRNA V3-4	Family decreased: Prevotellaceae; genus increased: *Bifidobacterium;* genus decreased: *Roseburia*	nd	sd
[92]	PD: 193 PSP: 22 MSA: 22 non-PD: 113	39 drug-naïve PD; age-, BMI-, region-matched; spouses as control; dietary habits assessed; autoimmune disease and advanced-stage PD excluded	16S rRNA V3-4	Family increased: Verrucomicrobiaceae, Enterobacteriaceae, Christensenellaceae, Lactobacillaceae, Coriobacteriaceae, Bifidobacteriaceae; family decreased: Lachnospiraceae; genus increased: *Akkermansia, Parabacteroides;* genus decreased: *Roseburia*	sd ( >)	sd
[93]	PD: 197 healthy: 103	Age 40–85 years, onset age 40–80 years, disease duration ≤ 12 years; age-matched; medications, diet, and demographics collected	16S rRNA V4	Family increased: Christensenellaceae, Desulfovibrionaceae; family decreased: Lachnospiraceae; genus increased: *Bilophila, Akkermansia;* genus decreased: *Roseburia, Faecalibacterium*	nd	sd
[94]	Training set PD: 40 healthy: 40 Validation set PD: 78 healthy: 75 MSA: 40 AD: 25	Spouses as partial control; no serious illness (e.g. heart failure); no chronic disease (e.g., diabetes); lifestyle factors and medications considered	shotgun metagenomic	Family increased: Carnobacteriaceae, Lactobacillaceae, Rikenellaceae, Streptococcaceae, Synergistaceae Genus increased: *Alistipes, Enterobacter, Gordonibacter, Granulicatella, Holdemania, Lactobacillus, Streptococcus* Species increased: *Clostridium_asparagiforme, Clostridium_leptum, Enterobacter_cloacae, Gordonibacter_pamelaeae, Granulicatella_unclassified, Holdemania_filiformis, Lachnospiraceae_bacterium 1_1_57FAA, Lachnospiraceae_bacterium 3_1_57FAA_CT1, Lactobacillus_salivarius, Paraprevotella_clara, Streptococcus_anginosus, Streptococcus_salivarius, Streptococcus_thermophilus*	sd ( >)	sd
[95]	PD: 26 Control: 25	Early, L-DOPA-naïve PD; only male; 11 healthy controls, 14 diseased controls had cardiovascular risk factors	shotgun metagenomic	Species increased: *Akkermansia muciniphila, Alistipes shahii, Alistipes obesi, Alistipes ihumii;* species decreased: *Prevotella copri, Clostridium saccharolyticum, Desulfibrio piger*	na	na
[96]	PD: 104 non-PD: 96	91 spouses, 5 siblings as control; diet, lifestyle and housing condition considered	16S rRNA V3-4	Family increased: Christensenellaceae, Verrucomicrobiaceae, Synergistaceae, Catabacteriaceae, Lactobacillaceae; genus increased: *Cloacibacillus, Catabacter, Christensenella, Butyrivibrio, Bifidobacterium, Megasphaera;* species increased: *Bacteroides fragilis, Lactobacillus acidophilus*	nd	na
[97]	PD: 490 healthy: 234	Region-matched; 55% controls were spouses	shotgun metagenomic	Genus: 23 ↑, 11 ↓; species: 55 ↑, 29 ↓	na	sd
[98]	PD: 96 non-PD: 74	Newly diagnosed PD; environmental factors considered; no immunocompromised	16S rRNA V4	Phylum increased: Proteobacteria, Verrucomicrobiota, Actinobacteria; genus increased: *Akkermansia, Enterococcus, Hungatella*	sd ( <)	sd

< , ↓: a lower abundance in patients with PD compared to controls; > , ↑: a higher abundance in patients with PD compared to controls

PD: Parkinson’s disease, nd: no difference, sd: significant difference, L-DOPA: Levodopa, BMI: body mass index, IBS: irritable bowel syndrome, RBD: rapid eye movement sleep behavior disorder, PSP: progressive supranuclear palsy, MSA: multiple system atrophy, AD: Alzheimer's disease, na: not available

**Fig. 1:**
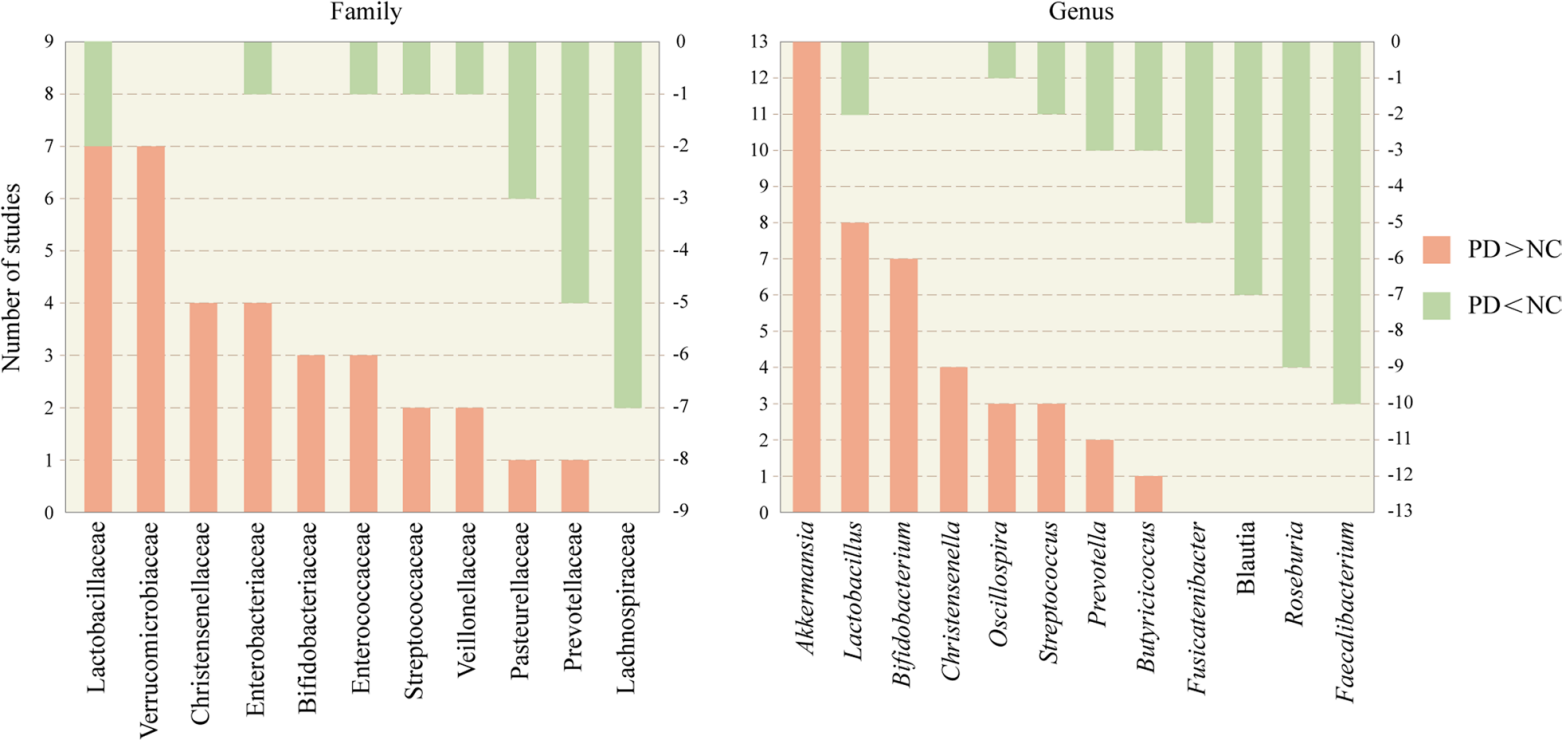
The most commonly reported 11 families and 12 genera of gut microbiota that are different between the PD and the NC groups. Orange bars represent the number of studies in which PD had a higher abundance than NC. Cyan bars represent the number of studies in which PD had a lower abundance than NC. PD, Parkinson’s disease, NC, normal control

**Fig. 2:**
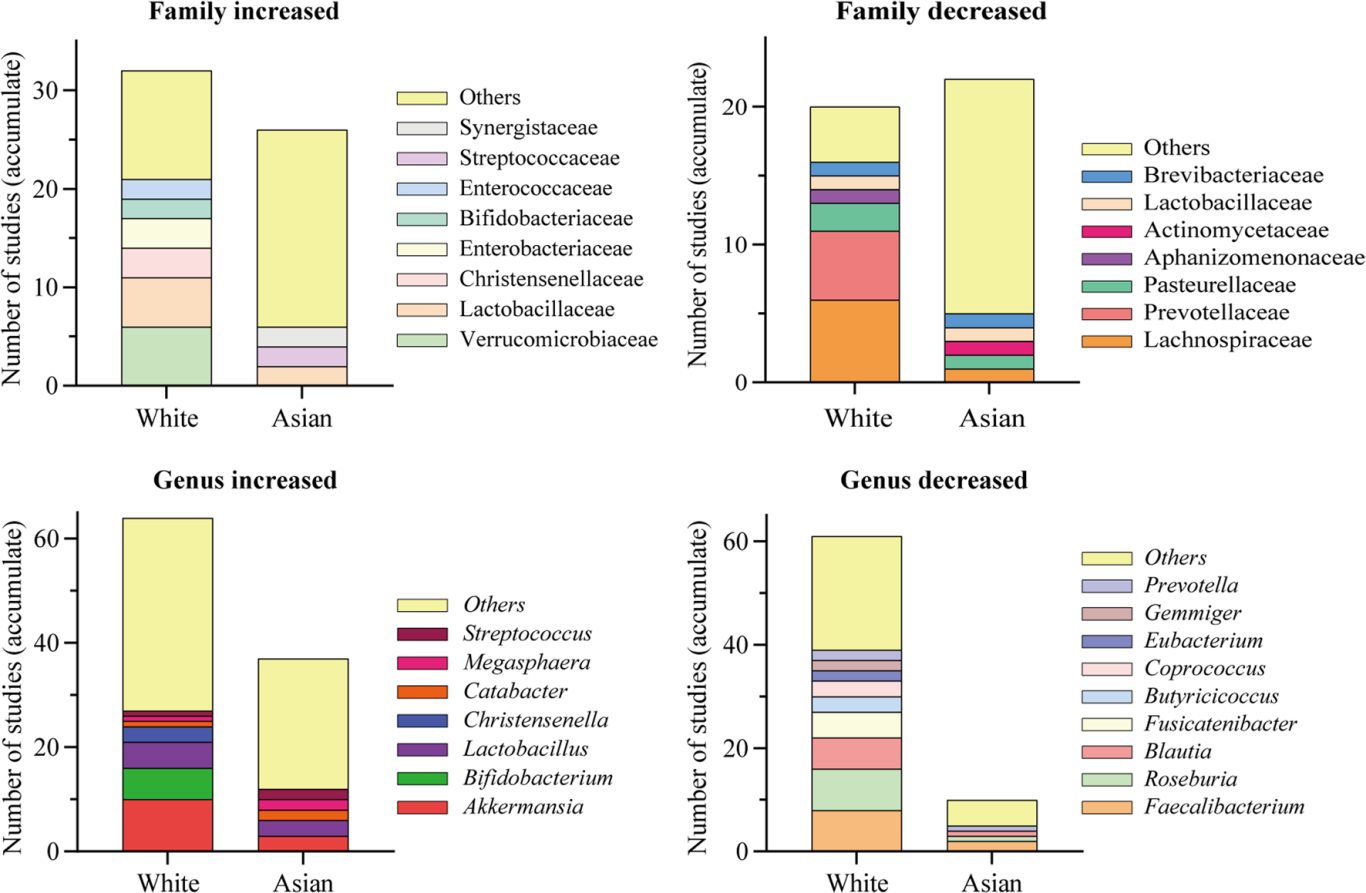
Alterations in intestinal flora in White and Asian populations with PD. The figure illustrates the number of times the intestinal flora at the family and genus levels have been cumulatively reported in the literature

Results regarding microbial alterations in the gut of PD patients vary; however, some findings have been robustly replicated (Fig. 1). For example, it has been consistently demonstrated that patients with PD show decreased *Lachnospiraceae* and *Prevotellaceae* abundances and increased *Verrucomicrobiaceae* and *Lactobacillaceae* abundances. It is also important to note that alterations in gut microbiology may vary across race/ethnicity, which may be related to factors such as genetic background, diet, environment, and the testing method used (Fig. 2) [86, 87, 89, 91, 92, 99–103]. Alterations in microbiota (e.g., increased abundance of *Christensenellaceae* or *Oscillospira*) are associated with an increased risk of developing PD, indicating that specific changes in the microbiome can be used to diagnose the disease at an early stage. Dysbiosis is already present in untreated patients with early-onset and treatment-naïve PD [100]. Surprisingly, the gut microbiota is altered in patients with idiopathic rapid eye movement sleep behavior disorder and this alteration has a similar trend to that of patients with PD, and is even already present in their first-degree relatives. This suggests that changes in gut microbiota have already occurred in the prodromal phase of PD [89, 104, 105]. The findings on gut microbes in the differential diagnosis of PD are inconsistent [92, 94, 106]. Therefore, using gut microbes as a biomarker for differential diagnosis of PD is premature.

### Gut microbiota and PD symptoms

The results of recent clinical studies indicate that gut microbes are associated with PD phenotypes, such as onset time [107], duration [100], disease stage [108], and clinical symptoms (both motor and nonmotor) [86, 89, 90, 92, 109]. Gut microbes may also help predict those who may be at risk for PD; for example, reduced abundance of short-chain fatty acid (SCFA)-producing microbes could predict the likelihood of future transition to PD in patients with idiopathic rapid eye movement sleep behavior disorder [105].

Regarding motor symptoms, abundance of *Lactobacillus* is correlated with the degree of impaired motor function [92], whereas abundance of the Enterobacteriaceae family is correlated with postural instability, walking difficulties, and akinetic-rigid subscores [86, 91]. Enterobacteriaceae, *Clostridium*, Verrucomicrobia, and *Akkermansia* levels help to distinguish whether PD is dominantly the tremor type [107, 110–112]. Besides motor symptoms, gut microbes may also be associated with nonmotor symptoms. Low counts of *Bacteroides fragilis* are associated with deterioration of motivation/activeness, whereas *Bifidobacterium* is correlated with hallucinations/delusions [113]. Based on data from a study of 423 patients with new-onset PD, gastrointestinal symptoms associated with gut microbiota dysregulation could predict cognitive function [114].

Despite advances in research on gut microbes and PD, there is poor consistency among the existing studies, most likely because gut microbes are influenced by numerous factors. Variations in experimental design (e.g., fecal collection methods, DNA extraction procedures, sequencing techniques, depth heterogeneity, and statistical methods), as well as different individual patient-related factors (e.g., geography, age, ethnicity, host genetics, diet, medications, lifestyle habits, disease severity, and other confounding factors) can affect the results. Therefore, reliable PD microbiome characteristics can only be obtained by adopting a rigorous study design, using standardized processes and methods, and using appropriate sample sizes. At the outset of the study, in addition to excluding subjects with acute and chronic gastrointestinal diseases and those who have recently taken antibiotics and probiotics that have a significant impact on intestinal microbiology, it is also necessary to consider the patient’s genetic background, disease stage, and severity of the condition, and match them with a control group by age, gender, geographical location, diet, underlying diseases, lifestyle, and environmental exposure. The interference of confounding factors such as comorbidities and medications should also be considered simultaneously [115–117].

## Gut microbial mechanisms in PD

**Fig. 3:**
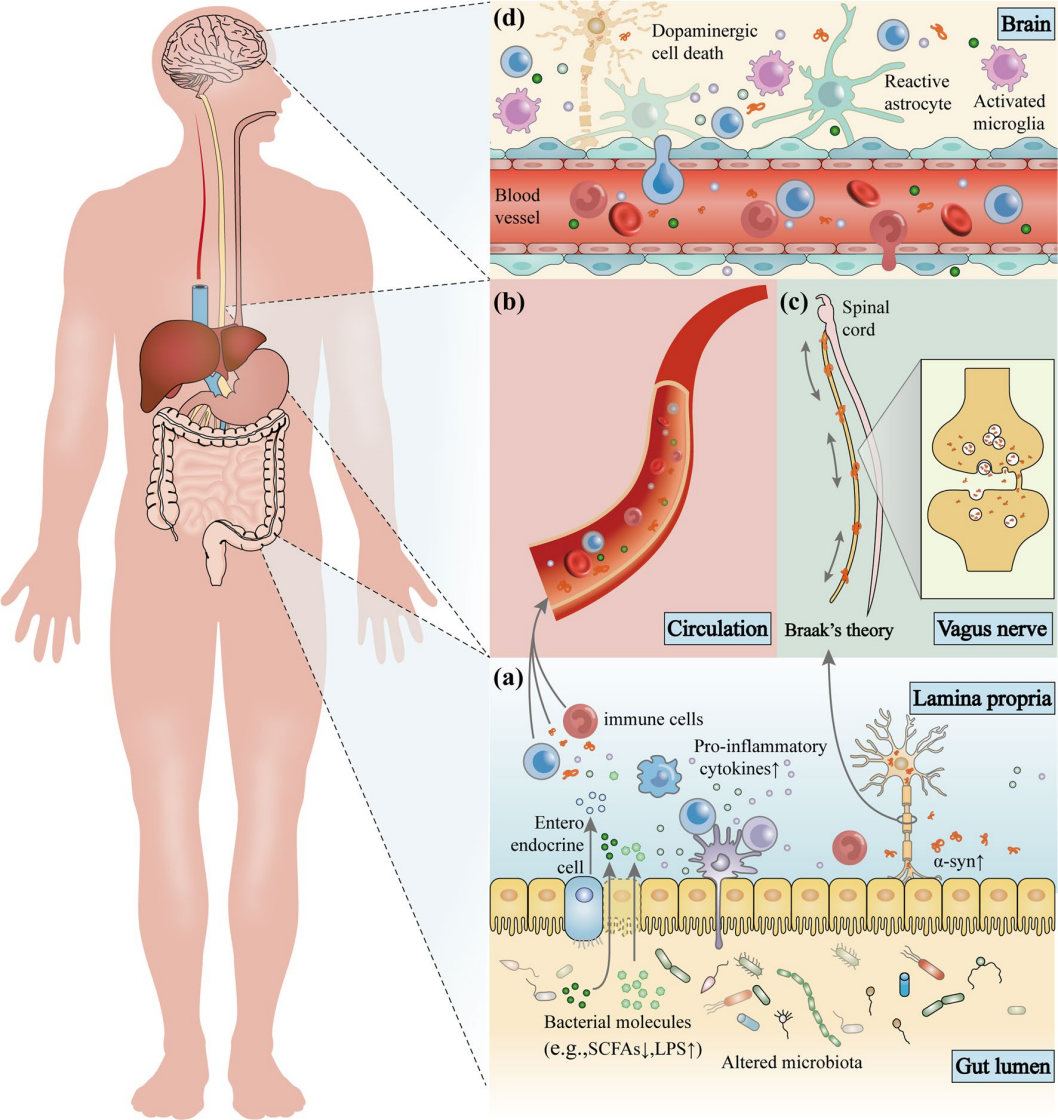
The microbiota–gut–brain axis in Parkinson’s disease (PD). Disordered gut microbes, through the microbiota–gut–brain axis, play a role in the pathogenesis of PD via the immune, endocrine, and nervous systems. **a** Alterations in intestinal microbes and their metabolites can leave the gut in an inflammatory state. These substances can cross the damaged intestinal barrier, activate mucosal immune cells, induce the release of pro-inflammatory cytokines, and promote misfolding and aggregation of α-syn. **b** Increased intestinal permeability allows release of signaling molecules by intestinal microbes and activated immune cells as well as through metabolic secretion to enter the circulation and cause systemic inflammation. **c** Misfolded α-syn in the gut can be transferred to the brain through intercellular transmission via the vagus nerve, and this transmission may be bidirectional. **d** The damaged blood–brain barrier and vagal pathways allow pathological products and α-syn to enter the brain, promoting the activation of immune cells in the brain, including microglia and astrocytes, causing neuroinflammation, and ultimately leading to the loss of dopaminergic neurons and the development of PD

The specific mechanisms underlying the relationship between gut microbiota and PD have not been fully elucidated, implicating multiple pathways that indirectly lead to dopaminergic neurodegeneration and various CNS dysfunction or impairment (Fig. 3) [118].

### Gut microbiota and α-syn

Gut microbes may lead to α-syn misfolding and PD pathology [100, 119]. Like prion disease, molecular mimicry-induced template cross-seeding may cause neuronal protein misfolding in PD [120]. The molecular mimicry of PD may be caused by an extracellular amyloid generated by gut bacteria [121–123]. For example, curli, an extracellular amyloid protein with structural and biophysical properties similar to human pathological amyloids, is secreted by *Escherichia coli* via coordinated biosynthetic processes. Curli may activate the innate immune system and facilitate α-syn aggregation and neuroinflammation [44, 124]. In animal experiments, addition of curli-producing bacteria to old Fischer 344 rats or α-syn-overexpressing *Caenorhabditis* *elegans* or mice induced accumulation of α-syn in intestinal and brain tissues, and this process could be reversed by depletion of curli through genetic or pharmacological approaches [44, 121, 125]. In another study, intraperitoneal injections of 1-methyl-4-phenyl-1,2,3,6-tetrahydropyridine (MPTP) in a mouse model increased α-syn expression in the ileal and affected gut microbiota composition [126]. In another mouse model overexpressing human α-syn, the absence of certain gut microorganisms reduced α-syn neuropathology, whereas the presence of the gut microbiota resulted in higher α-syn aggregation. Thus, genetically mediated α-syn overexpression and gut microbiota components have a combined impact on α-syn aggregation in the brain [45].

### Immunity and inflammation

Progressive dopaminergic neurodegeneration and substantial neuroinflammation in the striatal pathway of the substantia nigra (SN) are two signs of PD [64, 127]. Numerous findings suggest that the inflammation-induced oxidative stress and cytokine toxicity play important roles in PD [128–130]. Gut microbiota may regulate the inflammatory signals and interact with other organs, making it a key player in “inflammaging” [131].

#### Intestinal infection-related inflammation in PD

The elevated expression of inflammatory cytokine and chemokine genes in the intestinal tissues of patients with PD indicates that PD is closely associated with inflammation in the intestine [100, 132, 133]. Moreover, increased levels of glial cell markers (GFAP and Sox-10) and several pro-inflammatory cytokines are detected in colon biopsy tissues of patients with PD [133]. Similarly, increased amounts of various inflammatory mediators (e.g., interleukin [IL]-1β, IL-6, interferon gamma [IFN-γ], and tumor necrosis factor alpha [TNF-α]) in the stool samples of patients with PD indicate the presence of gastrointestinal inflammation [64, 119, 134–136]. These inflammatory changes increase the susceptibility of the host to immune dysfunction and autoimmunity [137].

#### Gut microbiota and intestinal inflammation

Gut microbial dysbiosis can result in peripheral and central immune activation and inflammation [138, 139], causing persistent intestinal epithelial inflammation and neuroinflammation via the microbiota–gut–brain axis [140, 141]. The blood–brain barrier (BBB) can be damaged by pro-inflammatory molecules in the systemic circulation, allowing inflammatory cytokines to enter the SN, leading to neuroinflammation and death of dopaminergic neurons [141–144].

Changes of anti-inflammatory bacteria have been detected in patients with PD. A study in PD patients revealed a considerable drop in *Blautia*, *Coprococcus*, and *Roseburia* genera in stool samples; a decrease of *Faecalibacterium* and increase of *Ralstonia* in the gastrointestinal mucosa; and a shift to a more inflammatory state of microorganisms in the colon [100]. The relative abundances of Verrucomicrobia and *Bacteroides* have been revealed in PD, which are associated with elevated plasma TNF-α and IFN-γ levels. This suggests that the gut flora is changed in a systemic sub-inflammatory condition in PD [109]. Notably, young *Pink1* knockout mice may develop severe dyskinesia and striatal dopaminergic axon loss later in life when exposed to Gram-negative bacteria that induce moderate intestinal symptoms, suggesting an interaction between a genetic predisposition to PD and intestinal microbes, as well as intestinal inflammation [145].

#### PD and inflammatory bowel disease (IBD)

Several systematic reviews and meta-analyses showed that individuals with IBD have a 20%–90% increased chance of developing PD [20–24]. In addition, individuals with IBD have fewer Firmicutes and more Enterobacteriaceae than healthy individuals [146]. These microbial characteristics of IBD are consistent with some microbial abnormalities identified in PD [101]. Research on IBD and PD has focused on numerous shared genetic risk factors [147], as the two diseases share several loci that influence the risks of both diseases in similar directions. IBD and PD are both associated with mutations of leucine-rich repeat kinase 2 (*LRRK2*), a gene that mediates microbial immunological signaling [25]. Substantial evidence supports the role of *LRRK2* in immune cells and inflammatory diseases [148, 149]. Recent animal studies have shown that colitis in *Lrrk2* p.G2019S mice is more severe than that in littermate controls or *Lrrk2* wild-type mice. The *Lrrk2* p.G2019S mice with colitis show reduced motor function and increased loss of dopaminergic neurons. *LRRK2* mutations significantly enhance inflammation in the colon and the brain and impact neuronal survival. This response is associated with immunomodulation by *LRRK2* [150, 151]. Similarly, increased *LRRK2* expression was observed in colon biopsies from patients with PD, and the expression levels correlated with disease severity, even in the prodromal phase of the disease, when colon *LRRK2* expression was dramatically increased [152]. *NOD2* is the most closely correlated gene for Crohn’s disease (CD) and has the most frequently replicated associations with CD. A meta-analysis showed that intronic single nucleotide polymorphisms (SNPs) of *NOD2* (rs6500328) are also related to the susceptibility to PD [153, 154]. In addition, patients with PD show an overexpression of the *CARD15* gene SNP, which is linked to CD [155]. There are also other reported risk loci for PD and IBD. These loci are involved in immune response and microbial induction (e.g., *HLA* locus) as well as in lysosomal dysfunction (e.g., *GALC* and *GPR65*), and are shared by PD and IBD with similar mechanisms [24, 49, 156]. The association between PD and IBD has also been confirmed therapeutically. A previous study showed that patients with IBD who received anti-TNF biologics as part of chronic anti-inflammatory treatment are 78% less likely to develop PD compared to those who did not, indicating that inhibiting peripheral inflammation may prevent PD [23, 25]. However, a subsequent Mendelian randomization research did not yield consistent results with those studies [27].

#### Toll-like receptors (TLRs)

TLRs are transmembrane pattern recognition receptor proteins that initiate the innate immune response by detecting foreign microbial and viral molecules and maintaining intestinal homeostasis [157]. Several studies have shown that PD is closely associated with TLRs. Patients with PD show elevated blood and brain levels of TLR2 and TLR4 [158]. Gut microbes influence TLR2 expression, and TLR2 recognizes bacterial products such as lipoteichoic acid, lipoproteins, peptidoglycans, and bacterial amyloid (e.g., curli protein) [159]. By binding and activating TLR2, curli increases intracellular α-syn, triggering a neuroinflammatory response via the TLR2/MyD88/NF-κB pathway [160, 161]. Similarly, activation of TLR2 in the brains of PD patients increases proinflammatory cytokine levels and microglial recruitment and amplifies neuroinflammation and α-syn expression. Most α-syn-positive Lewy bodies also exhibit high TLR2 immunoreactivity, indicating a strong relationship between these pathologies [162, 163]. Similarly, more TLR4-expressing cells are observed in the colonic tissues of PD patients than in healthy controls [164]. The TLR4 signaling system, which recognizes Gram-negative bacterial lipopolysaccharides (LPS) and endogenous chemicals, plays a significant role in the inflammation observed in the intestines and brains of patients with PD [132]. TLR4 is crucial for clearing α-syn; in addition, it interacts with α-syn to initiate PD-associated microglial cell responses [165, 166]. TLR4 deficiency significantly attenuates the effects of rotenone on intestinal barrier integrity, GFAP expression in myenteric plexuses, colonic α-syn, nigrostriatal microglial activation, dopaminergic neuron loss, and motor dysfunction [132]. Considering the crucial role of TLR4 in PD, studies on the treatment of PD by targeting and modulating this pathway have been conducted, and the results imply that the microbiota–gut–brain axis is involved [167].

### Microbial toxins: LPS

Gram-negative bacteria produce the endotoxin LPS in their cell walls. PD-associated intestinal dysbiosis results in LPS-mediated intestinal inflammation and weakens the intestinal barrier by activating the TLR4/MyD88/NF-κB signaling cascade [109].

Peripheral injection of LPS in C57BL/6J mice increases pro-inflammatory cytokine levels and activates microglia in the SN via the NOD-like receptor protein 3 (NLRP3)–IL-1β signaling pathway, leading to brain neurodegeneration [168]. In CNS disorders, the NLRP3 inflammasome is essential for the intestinal/peripheral inflammation caused by microbiota and neuroinflammation [169]. Moreover, another study showed that repeated administration of *Proteus mirabilis* or LPS to young wild-type or MPTP-treated mice is sufficient to cause symptoms similar to PD [170, 171]. In addition, LPS can facilitate the transfer of α-syn to the CNS by binding to it and initiating intestinal fibrosis [172, 173]. This finding supports the hypothesis that the gut microbial pro-inflammatory environment is a major factor in the pathogenesis of PD.

### Gut microbiota-derived metabolites and PD

Changes in microbial metabolites can modulate CNS pathophysiology. Gut microbes produce approximately 40% of human metabolites, including SCFAs, trimethylamine N-oxide (TMAO) and amino acids, which have several physiological functions [174]. Metabolic condition appears to be more important than species balance in terms of microbiome function [175]. Alterations in the composition of the microbiota may lead to metabolic changes in PD, which may play an important role in the onset and progression of PD, with SCFAs being particularly important (Table 3) [176].

**Table 3:** Altered gut microbial metabolites in patients with PD

Ref.	Sample size	Sample	Control factors	Dietary instruments	Technique	Findings
[101]	PD: 34 Healthy: 34	Fecal	Age-matched; no special dietary habits	Dietary habits were interviewed	Gas chromatography	PD is associated with certain gut microbiota and reduced fecal SCFAs
[93]	PD: 75 Healthy: 50	Serum	Aged 40–85 years, onset age 40–80 years, disease duration ≤ 12 years; age-matched; medications, diet, and demographics collected	FFQ	UPLC-MS; HILIC-MS	The microbiota of PD had decreased carbohydrate fermentation and butyrate synthesis and enhanced proteolytic fermentation and p-cresol and phenylacetylglutamine production. Patients with constipation and stool consistency had more proteolytic metabolites and taxonomic changes
[206]	PD-MCI: 13 PD-NC: 14 Healthy: 13	Fecal	Spouses as control; age-matched; BMI-matched; no serious chronic illnesses (e.g., hyperlipidemia, diabetes); no fat-rich diet	Questionnaire including caffeine and alcohol intake	GC–MS	SCFAs were similar in PD-MCI, PD-NC, and healthy, however, the isovaleric and isobutyric levels negatively correlated with the MMSE scores
[194]	PD: 64 Healthy: 51	Fecal	Spouses or family members as control; internal medicine, neurological, or unstable psychiatric illness excluded	N.A	GC–MS	Lipids, vitamins, amino acids, and other organic compounds changed. Most modified metabolites closely associated with *Lachnospiraceae* abundance
[95]	PD: 8 Control: 10	Serum	Early, L-DOPA-naïve PD; only male; 5 healthy controls, 5 diseased controls having cardiovascular risk factors	Omnivorous vegetarian probiotics	Targeted metabolomics	Disease severity is linked to mucin and host glycans breakdown by microbes. Gut-community metabolic modeling shows that PD bacteria cause folic acid deficiency and hyperhomocysteinemia
[96]	PD: 104 Non-PD: 96	Fecal	91 spouses, 5 siblings as control; diet, lifestyle and housing condition considered	FFQ	NMR; LC–MS	Neuroprotective chemicals such as SCFAs, ubiquinones, and salicylate, as well as ceramides, sphingosine, and TMAO, are linked to PD metabolite features and functional changes. Clinical signs include cognitive impairment, BMI, frailty, constipation, and physical activity are also linked to it

PD: Parkinson’s disease, SCFAs: short chain fatty acids, FFQ: Food Frequency Questionnaire, UPLC-MS: ultraperformance liquid chromatography-mass spectrometry, HILIC-MS: hydrophilic metabolites for hydrophilic interaction liquid chromatography-mass spectrometry, MCI: mild cognitive impairment, N.A.: non-available, NC: normal cognition, BMI: body mass index, GC–MS: gas chromatography-mass spectrometry, MMSE: Mini-Mental State Examination, na: not available, L-DOPA: Levodopa, NMR: nuclear magnetic resonance, LC–MS: liquid chromatography-mass spectrometry, TMAO: trimethylamine N-oxide

#### Two sides of SCFAs

SCFAs are significant byproducts of intestinal microorganisms and mainly include formic, acetic, propionic, and butyric acids. Patients with PD show significantly decreased levels of acetate, propionate, and butyrate in stool samples [177]. Butyrate can provide energy to the intestinal epithelial cells. In addition, it suppresses the activity of the transcription factor NF-κB and decreases intestinal mucosal inflammation, which modulates the action of pro-inflammatory cytokines [178, 179]. Moreover, butyrate may activate autophagy via the Atg5 and PI3K-Akt-mTOR pathways, leading to degradation of α-syn in rat models of pesticide-induced PD. Animal studies have demonstrated that some SCFAs have significant protective effects on striatal dopaminergic and tyrosine hydroxylase-positive neurons [180–182]. Changes in the gut microbiota-mediated SCFAs may be a driving force for dopaminergic neuronal degeneration. Reduction of several SCFA-producing bacteria, such as *Roseburia, Eubacterium*, *Ruminococcus*, *Blautia*, *Faecalibacterium prausnitzii*, and *Coprococcus*, many of which are also butyric acid-producing bacteria, has been observed in PD [97, 100, 101]. Reduction of gut microbiota-derived SCFAs leads to decreased colonic motility and mucin synthesis as well as increased intestinal mucosal inflammation and permeability [38, 183–185]. This exposes the internal environment to bacterial antigens and endotoxins, causing systemic inflammation, neuroinflammation and neurodegeneration, and leading to overexpression, misfolding, and reduced clearance of α-syn, thus promoting PD dyskinesia [45, 186]. Conversely, increasing the gut bacteria that produce butyric acid can protect the damaged intestinal barrier and increase the striatal dopamine level [187]. Butyric acid, produced by the gut microbiota when prebiotic fiber is fermented, is regarded as a promising treatment option for PD [188, 189]. In an animal study, sodium butyrate, an HDAC inhibitor, ameliorated dyskinesia in a *Drosophila* PD model [182]. In another study, long-term administration of phenylbutyrate increased DJ-1 activity, decreased α-syn aggregation, and prevented age-related motor deterioration and cognitive impairment in a transgenic mouse model of diffuse Lewy body disease [190]. Recent studies have found that fermentation of feces from PD patients with prebiotic fibers can alter the microbiota and promote production of SCFAs that may exert effects on microglia indirectly [191, 192].

Despite the reported benefits, the position of SCFAs in PD is debated. Recent studies have shown no change or even an increase in SCFAs in patients with PD [193, 194]. Interestingly, systemic SCFAs levels are raised even when the fecal levels decrease in patients with PD, possibly due to the impaired gut-blood barrier permeability that may allow SCFAs to enter the systemic circulation [195]. Animal studies similarly found that the increased SCFAs in the feces of mice treated with MPTP were associated with increases of activated striatal glial cells, including microglia and astrocytes [196]. Sampson et al. [45] showed that supplementing SCFAs to germ-free (GF) mice promotes α-syn-mediated neuroinflammation and motor deficits. It is important to note that providing SCFAs to GF animals (which produce little to no SCFAs) results in an acute inoculation state that dramatically alters host physiology and matures immune function [197, 198]. Similarly, pathology has been observed in other neurodegenerative GF model mice and microbe-free human cell culture systems [199–201]. In other research, sodium butyrate accelerates motor dysfunction and dopaminergic neuron death in PD mice. It downregulates the dopamine level and increases the numbers of activated microglia and astrocytes, increasing the glial cell-mediated neuroinflammation [202].

The SCFAs and SCFA-producing bacteria are not disease-specific, and the causal link between SCFAs and various PD pathologies is currently unclear. Further evidence is needed to determine whether SCFAs modulate specific neurons directly [25]. SCFAs may have both positive and negative influence on the autoimmune CNS inflammation [203], and depending on the dose and type, different SCFA concentrations and ratios may result in different health outcomes [204]. The inconsistency of results among the SCFA studies calls for the need to consider possible influences from environment (GF vs. SPF), diet, genetic background, disease stage, form of intervention, and assay methods in animal and clinical studies in the future [192, 201, 205].

#### TMAO

TMAO is a metabolite derived from intestinal microorganisms and synthesized from dietary elements such as* L*-carnitine and choline [207]. TMAO disrupts the BBB and alters the NLRP3 inflammasome, which may contribute to neuroinflammation and PD. Additionally, TMAO may stimulate human α-syn folding in a dose-dependent manner [208]. In mice, TMAO may cause oxidative stress, neuronal senescence and synaptic impairment, leading to brain aging [209]. However, there is also evidence that TMAO may be neuroprotective by facilitating proper protein folding and reducing endoplasmic reticulum stress and α-syn formation [210]. Clinical findings on TMAO are also inconsistent. One study found elevated TMAO to be associated with worsening of motor symptoms and dementia transformation in PD; conversely, decreased TMAO has also been reported to be associated with PD progression and dementia transformation [96, 210, 211]. The contradictory results may be attributed to the confounding bias or reverse causality. Recent research has shown a high link between cerebrospinal fluid and plasma TMAO levels, supporting that peripheral TMAO may enter the CNS [212].

#### Amino acids

The gut microbiota is critical for amino acid metabolism and cycling, and amino acid-fermenting bacteria can regulate amino acid distribution in the gastrointestinal tract [194]. Branched-chain amino acids (BCAAs) and aromatic amino acids (AAAs) in the body are mainly derived from dietary nutrients; therefore, gastrointestinal dysfunction in PD may impair their absorption [213]. It has been reported that the disturbances in plasma BCAAs and AAAs in PD patients may be related to the gut microbiota [214]. BCAAs regulate brain function. Tyrosine and phenylalanine produced during AAA metabolism are key substrates for the production of dopamine. Tryptophan is processed by host cells and some intestinal bacteria to serotonin, kynurenine, and indole derivatives that act as neurotransmitters and metabolic regulators [215, 216]. Tryptophan and kynurenine levels are considerably lower in PD patients, suggesting the involvement of this pathway in PD etiology [217]. Notably, the findings of various research on BCAA and AAA alterations in PD have been conflicting [214, 218–220]. Therapeutically, a high-BCAA diet inhibits the pro-inflammatory state in the gut and the brain of mice, restores gut and motor function, and attenuates the loss of dopaminergic neurons [221]. Similarly, tryptophan supplementation prevents the rotenone-induced neurotoxicity and improves motor impairments, perhaps via the aromatic hydrocarbon receptor pathway [222]. Thus, amino acid supplementation may be a promising therapeutic target for PD.

### Neuroprotective factors and gut microbiota: ghrelin

Ghrelin is a signaling peptide involved in the gut–brain axis. It interacts with the CNS indirectly via the vagus nerve or directly across the BBB, triggering its target receptor GHSR-1a, which is present in various peripheral and brain areas. Interestingly, the ghrelinergic system and the gut microbiota have synergistic effects in controlling metabolic and central homeostatic functions [223, 224]. Increasing evidence supports the association between ghrelin disturbance and PD, and gut microbe may mediate this disturbance. Ghrelin and ghrelin receptors have significant neuroprotective effects in PD [225–231], and a dramatic decrease of their concentrations may be involved in the pathogenesis of PD [232]. Injection of the GHSR-1a antagonist [D-Lys3]-GHRP6 into the SN zone of normal mice triggers PD-like dyskinesia [232]. Patients with PD exhibit reduced plasma ghrelin levels [233], which are linked to increased Lactobacillaceae and decreased Prevotellaceae levels [234]. Ghrelin protects dopaminergic neurons by decreasing α-syn accumulation and phosphorylation, increasing autophagy, and blocking the endoplasmic reticulum-mediated apoptosis [235]. *GHSR* gene deletion dramatically enhances the degeneration of dopaminergic neurons, leading to an abrupt decrease in dopamine concentration in the striatal region [225].

Ghrelin may be a novel and efficient therapeutic option for PD [236–238]. Ghrelin-assisted treatments can significantly increase the number of midbrain neural stem cells that promote dopaminergic nerve cell differentiation through the Wnt/β-catenin pathway [239]. In addition, ghrelin and its agonists promote gastric emptying and increase plasma levels of levodopa (*L*-dopa) and dopamine, which may be utilized for alleviating gastrointestinal problems that appear after PD and *L*-dopa treatment [237].

## Reflections on animal models of gut microbes in PD

Animal model experiments allow for control over several parameters such as host genetics, ambient circumstances, nutrition, chronobiological measures, gut microbiota, and regional/mucosal sampling [240]. Mechanistic investigations of the gut microbiota in PD have made some headway with the use of animal models (Table 4). However, to date, there is no animal model that can comprehensively encompass all the pathogenic features of PD.

**Table 4:** Mechanistic studies of microbiota in animal models of PD

Ref.	Animal model	Perturbation	Control factors	Test (phenotype and pathology)	Outcomes	Summarize
[45]	Thy1-α-syn mice	GF versus SPF	Housed in sterile or autoclaved caging, receiving autoclaved food	Beam traversal, pole descent, nasal adhesive removal, hindlimb clasping reflex, α-syn inclusions, microglia morphology	Gut microbiota promotes α-syn-mediated motor impairments and brain damage; depletion of gut bacteria reduces microglial activation; SCFAs regulate microglia and exacerbate PD pathophysiology; in mice, gut microbiota from PD patients enhances motor impairment	Gut microbes may play a key functional role in the pathogenesis of PD
[145]	*Pink1*^−/−^ mice	Administration of *Citrobacter rodentium*	Littermate mice, kept in pathogen-free conditions	Behavioural tests, grip strength test, basal locomotor activity, pole test, histology for dopaminergic neurons	*Pink1*^−/−^ mice with intestinal infection exhibited dyskinesia; significant reduction in dopaminergic axonal varicosities; mitochondria-specific CD8^+^ T cells in the brains of infected *Pink1*^−/−^ mice killed dopaminergic neurons in vitro	Supports PINK1 as an immune system suppressor and implies that intestinal infections may induce PD
[241]	*Caenorhabditis elegans*	*Bacillus subtilis* probiotic strain PXN21 feeding	All strains were grown at 20 °C, bacteria were grown in SSM medium at 37 °C for 48 h	Locomotion analysis, lifespan assays, quantification of life-traits, α-syn forms and expression levels, nematode RNA sequencing	*Bacillus subtilis* PXN21 inhibits and reverses α-syn aggregation in a *Caenorhabditis elegans* model; probiotics alter host sphingolipid metabolism, whereas gut biofilm formation and bacterial metabolites diminish α-syn aggregation	A foundation for exploring the disease-modifying potential of *Bacillus subtilis* as a dietary supplement
[242]	Rotenone mouse model	GF versus CR	Age- and weight-matched, under sterile conditions	Grip strength test, rotarod test, intestinal permeability measurement, quantification of TH neurons	Rotenone gavage caused TH neuron loss in GF and CR mice, but only CR mice had impaired motor strength and coordination; rotenone affected intestinal permeability in CR mice but not GF animals	The gut microbiota has a potential role in modulating barrier dysfunction and motor deficits in PD
[243]	MPTP- mouse model	Administration of Cb	Animals were kept at 23 ± 2 °C with 12 h light/dark cycles	Pole test, beam walking teat, forced swimming test, open field test, dopaminergic neuron loss, synaptic plasticity, microglial activation	Oral administration of Cb ameliorates MPTP-induced motor deficits, dopaminergic neuron loss, synaptic dysfunction, and microglial activation in mice	Cb exerts neuroprotective effects by modulating the abnormal microbiota-gut-brain axis
[244]	Rotenone mouse model	Administration of *Lactobacillus plantarum* PS128	Under standard laboratory conditions	Rotarod test, narrow beam test, dopamine level, quantification of TH neurons, microglial activation, neuroinflammation	PS128 dramatically improved motor impairments in PD-like animals by increasing brain dopamine levels, neurotrophic factor expression, decreasing dopaminergic neuron loss, microglial activation, inflammatory factors	By modulating gut microbiota, PS128 improves motor function and neuroprotection in PD
[192]	Thy1-α-syn mice	Feeding a prebiotic high-fiber diet	Housed in sterile, autoclaved cages with sterile water	Beam traversal test, pole test, wire hang, hindlimb score, adhesive removal, fecal output, microglia isolation and sequencing, immunohistochemistry, α-syn aggregation, flow cytometry, gut microbiome profiling	Prebiotic diet improves gut flora, lowers motility abnormalities, and reduces α-syn aggregation in the substantia nigra, mediated by microglia. Prebiotic diet decreases microglial activation and boosts disease resistance. Depletion of microglia reduces prebiotic benefits	Gut microbiome digestion of dietary fiber changes CNS cell physiology and improves behavioural and pathologic outcomes
[44]	Aged male Fischer 344 rats; α-syn-expressing *C. elegans*	Exposed to curli-producing bacteria	Rats: antibiotic treatment; *C. elegans:* standard conditions	Swimming tests, α-syn accumulation and aggregation, inflammation	Exposure to curli-producing bacteria in rats showed increased α-syn deposition in the gut and brain, increased microgliosis and astrogliosis, and elevated brain TLR2, IL-6, and TNF expression. α-syn-expressing *C. elegans* fed with curli-producing bacteria showed increased α-syn aggregation	Amyloid proteins in the microbiota have a role in the onset and progression of neurodegenerative illness
[171]	MPTP/p, MPTP, 6-OHDA-induced mice	Administration of *P. mirabilis*	Conditions: 23 ± 1 °C, relative humidity 60% ± 10%, 12 h light/dark cycle	Pole test, open field test, rotarod test, dopaminergic neuronal damage, activated microglia, LPS levels, colonic pathology, α-syn filament quantitation, α-syn expression	Administration of *P. mirabilis* significantly induced motor impairments, dopaminergic neuron loss, and inflammation in the substantia nigra and striatum and increased α-syn aggregation in the brain and colon	*P. mirabilis* may have a role in the etiology of PD
[245]	6-OHDA rat model	Antibiotic treatment	Conditions: 22 °C, 12/12 h light/dark cycles	Cylinder test, forepaw stepping test, amphetamine-induced rotation test, quantification of DA, its metabolites, and 5-HT, [^3^H]-DA uptake, DA neuron depletion, TH immunoreactivity, DAT expression and function, pro-inflammatory markers	Antibiotics decreased motor impairments, TH loss in the striatum and substantia nigra, and pro-inflammatory cytokines	Expands knowledge of gut microbiota’s function in DA neuronal vulnerability, motor behavior, and neuroinflammatory responses in PD
[125]	Thy1-α-syn mice	Colonization with curli-producing gut bacteria	Housed in sterile or autoclaved caging, receiving autoclaved food	Beam traversal, pole descent, fecal output, wire hang, adhesive removal and hindlimb scoring, α-syn pathology, inflammatory responses, microglia morphologies	Gut exposure to bacterial amyloid worsens motor impairments and α-syn brain disease via CsgA aggregation	These findings reveal a trans-kingdom link between the gut microbiome and mammalian amyloids, implying that some bacterial taxa may worsen neurologic illness
[242]	Rotenone mouse model	GF versus CR	Age/gender-matched GF mice were treated under sterile conditions	Grip strength test, rotarod test, quantification of TH neurons, intestinal permeability measurement	Chronic rotenone treatment disrupts colonic epithelial permeability and causes motor symptoms exclusively in CR mice with complex microbiota but not in GF mice	Demonstrate that gut microbiota may regulate PD barrier dysfunction and motor impairments
[121]	*C. elegans*	Feeding with *E. coli* knockout mutants	*C. elegans* were maintained at 20 °C	*C. elegans* basal slowing response assays, *C. elegans* butanone associative learning assays, cell viability assay, mitochondrial respiration assay, level of α-syn, the colocalization between CsgA and α-syn	Genetically deleting or pharmacologically suppressing the curli main subunit CsgA in *E. coli* lowered α-syn-induced neuronal mortality, increased mitochondrial health, and enhanced neuronal functioning. Through cross-seeding, CsgA colocalized with α-syn within neurons and enhanced its aggregation	Bacterial components (e.g., curli) can directly affect neurodegenerative lesions
[246]	MPTP- mouse model	FMT from healthy mice	Kept at 22–26 °C, 12 h light/dark cycle	Pole test, traction test, SCFAs analysis, α-syn expression, TH level, microglial marker, neuroinflammation	FMT improved physical function and lowered fecal SCFAs. FMT also reduced the expression of α-syn, prevented microglial activation in the SN, and hindered TLR4/PI3K/AKT/NF-κB signaling in the SN and striatum	FMT may protect mice against PD by reducing α-syn expression and inactivating TLR4/PI3K/AKT/NF-κB signaling
[247]	MPTP- mouse model	FMT from PD patients or healthy human controls	Conditions: 21 ± 1 °C, humidity 55% ± 5%, 12 h light/dark cycle	Pole test, rotarod test, gut inflammation, phosphorylated AMPK and SOD2 expression, TH expression, glial activation, CD13, PDGFRβ, CD31	FMT derived from healthy human controls may repair gut dysbacteriosis and improve neurodegeneration by suppressing microgliosis and astrogliosis, improving mitochondrial deficits via the AMPK/SOD2 pathway, and restoring nigrostriatal pericytes and BBB integrity	Human gut microbiota changes may be a risk factor for PD, and FMT may be used for preclinical therapy

GF: germ-free, SPF: specific-pathogen-free, α-syn: alpha-synuclein, SCFAs: short chain fatty acids, PD: Parkinson's disease, SSM: Schaeffer’s sporulation medium, CR: conventionally raised, TH: tyrosine hydroxylase, MPTP: 1-methyl-4-phenyl-1,2,3,6-tetrahydropyridine, Cb: Clostridium butyricum, *P. mirabilis*: *Proteus mirabilis*, 6-OHDA: 6-hydroxydopamine, DA: dopamine, DAT: dopamine transporter, 5-HT: 5-hydroxytryptamine, *E. coli*: *Escherichia coli*, FMT: fecal microbiota transplantation, SN: substantia nigra, BBB: the blood–brain-barrier

The animal models of PD are primarily divided into neurotoxic and genetic models. Neurotoxin-based models display degeneration of dopaminergic neurons in the substantia nigra pars compacta, but this model lacks the formation of Lewy bodies, the primary pathological hallmark of PD. In contrast, genetic animal models are typically dissimilar to the human condition and rarely reproduce the general traits of the disease [248]. Furthermore, the aging process differs significantly amongst various species of animals. Rodents may lack normal neurodegeneration due to their short lifetime [249]. Although mouse models may replicate the protein misfolding and aggregation found in human brains, most mouse models fail to fully recapitulate the symptoms and pathology of neurodegenerative disorders. The human gut microbiota, on the other hand, is complicated, with high inter-individual variations and numerous confounding factors. Animal models have severe limitations in terms of experimental control, scalability, and recapitulation of human gut interactions with host-specific symbionts and pathogens [250, 251]. Experimental microbial communities may not accurately match the human microbiome, and the gut microbiota of animal models may differ between laboratories [252]. Animal models have substantially improved our knowledge of the molecular involvement of gut microbiome in many illnesses, but their experimental findings are still far from clinically applicable.

## Gut microbes and PD drugs

PD medication therapies may be affected by gut bacteria [253–255]. The trillions of microorganisms comprising the gut bacteria produce various enzymes that can directly alter and metabolize drugs, affecting their bioavailability and efficacy [256, 257].

### Levodopa

**Fig. 4:**
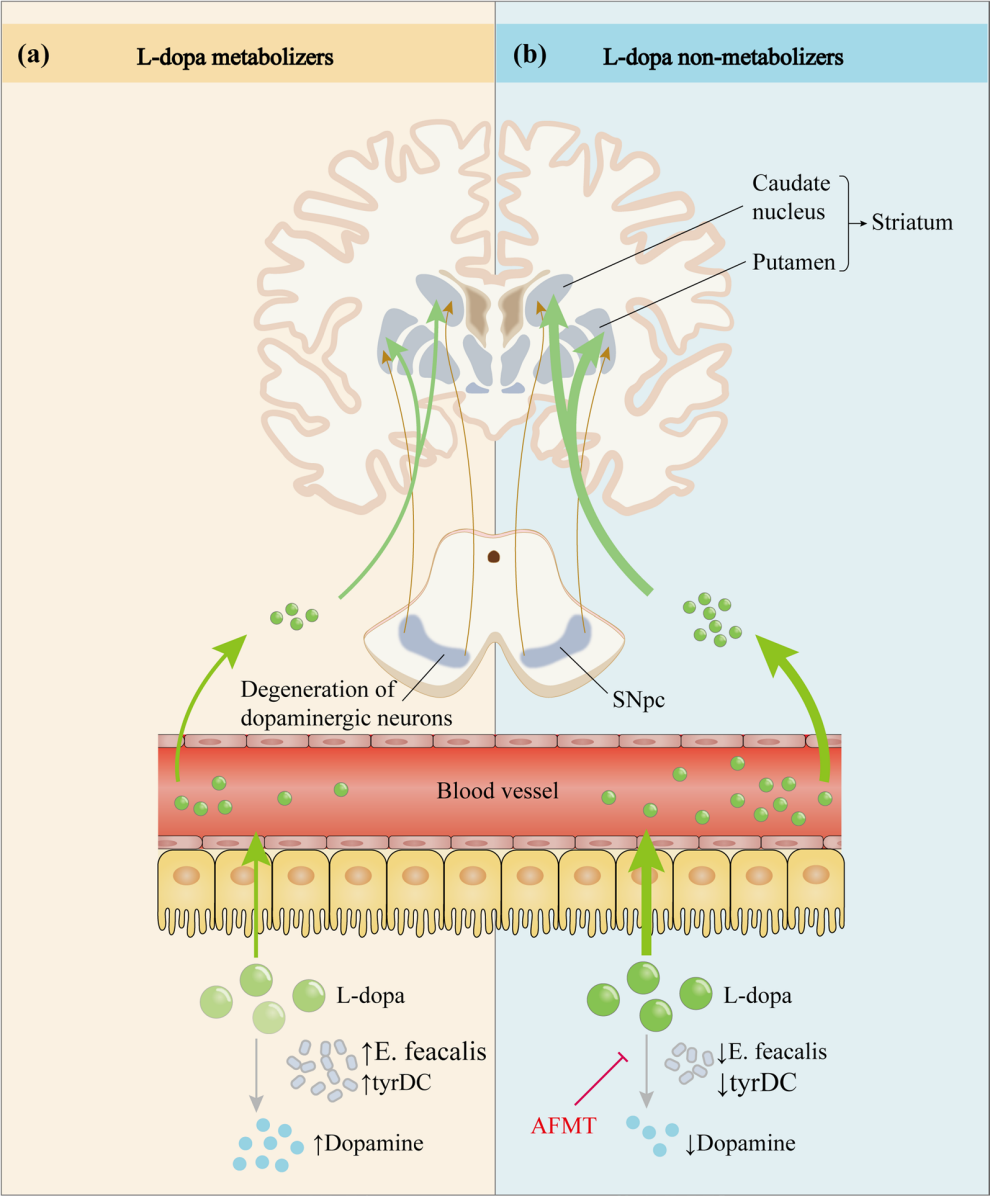
Effect of intestinal microbes on the metabolic pathway of levodopa. After oral administration, *L*-dopa enters the circulation through active transport in the intestine and crosses the blood–brain barrier into the brain, where it exerts anti-Parkinson’s disease effects by restoring striatal dopaminergic neurotransmission. However, only a small fraction of the drug eventually reaches the brain due to interference by various factors. Studies have revealed that tyrDC from *Enterococcus faecalis* can convert* L*-dopa to dopamine in the intestine and affect its absorption. **a** Elevated *E. feacalis* and tyrDC levels enable more *L*-dopa to be metabolized to dopamine in the intestine, resulting in impaired *L*-dopa absorption. **b** Conversely, a decrease in tyrDC allows more *L*-dopa to be absorbed and utilized. In addition, the small molecule inhibitor (S)-α-fluoromethyltyrosine (AFMT) can suppress tyrDC, thereby increasing the bioavailability of *L*-dopa

*L*-dopa is the most clinically used anti-PD drug [258]; however, its bioavailability varies greatly among patients. Given that *L*-dopa is usually administered orally or enterally, scientists believe that gut bacteria may impact its efficacy [87, 259, 260]. van Kessel et al. [255] and Maini Rekdal et al. [253] proved that gut microbiota can regulate *L*-dopa metabolism and they identified a two-step enzymatic pathway for *L*-dopa metabolism by intestinal microbes. First, a pyridoxal phosphate-dependent tyrosine decarboxylase (tyrDC) from the gut microbiota transforms *L*-dopa into dopamine, which is then changed into *m*-tyrosine by a molybdenum-dependent dehydroxylase from *Eggerthella lenta*. Analyses of established human microbiome datasets revealed that the *tyrDC* gene is mostly present in the *Enterococcus* and *Lactobacillus* genera, particularly in *Enterococcus*. The relative abundance of *tyrDC* gene in the fecal microbiota of PD patients was positively associated with higher daily levodopa/carbidopa dosage requirement and disease duration, suggesting that gut microbes can influence the efficacy of PD medications. Further studies in rats that received oral levodopa/carbidopa revealed that the plasma *L*-dopa levels are negatively correlated with jejunum bacterial *tyrDC* gene abundance, suggesting that overexpression of the bacterial *tyrDC* gene leads to detrimental *L*-dopa metabolism in the intestine [255]. In addition, researchers also discovered a small-molecule inhibitor, (S)-α-fluoromethyltyrosine (AFMT), that specifically inhibits *L*-dopa decarboxylation by tyrDC, *Enterococcus faecalis*, and gut microbiota samples from patients with PD, indicating that AFMT may boost *L*-dopa serum concentrations and increase the amount of *L*-dopa entering the brain, thereby improving its bioavailability (Fig. 4) [253].

These results imply that gut microbiota species and their enzymes are promising biomarkers for predicting the efficacy of *L*-dopa therapy, as they can affect the bioavailability and efficacy of *L*-dopa in vivo and provide a novel treatment strategy for PD.

### Other drugs

Catechol-O-methyl transferase (COMT) inhibitors are often used for the treatment of PD. Studies of gut microbes have revealed an association between COMT inhibitors and alterations in specific taxa; however, the results of these studies are not consistent [91, 101, 102, 108]. Entacapone, a well-established COMT inhibitor, inhibits the growth of *Faecalibacterium prausnitzii* and its potential bioactive metabolite butyrate [101]. Scheperjans et al. [86] discovered a correlation between COMT inhibitors and the abundance of Enterobacteriaceae. Patients treated with COMT inhibitors show a higher abundance of *Bifidobacterium* than those who did not receive the treatment [91]. However, some studies have shown that COMT inhibitors and anticholinergics can also lower the level of *Bifidobacterium* [102]. In addition, the gastrointestinal adverse effects of COMT inhibitors may be associated with microbiota dysbiosis [261].

Dopamine agonists (pramipexole and ropinirole) significantly decrease the small intestinal motility and increase the distal small intestinal bacterial overgrowth in rats. These microbial changes include increases in the abundance of *Lactobacillus* and *Bifidobacterium* and decreases in the abundance of Lachnospiraceae and Prevotellaceae. These findings are consistent with those observed in humans [262].

## Microbial therapy

**Fig. 5:**
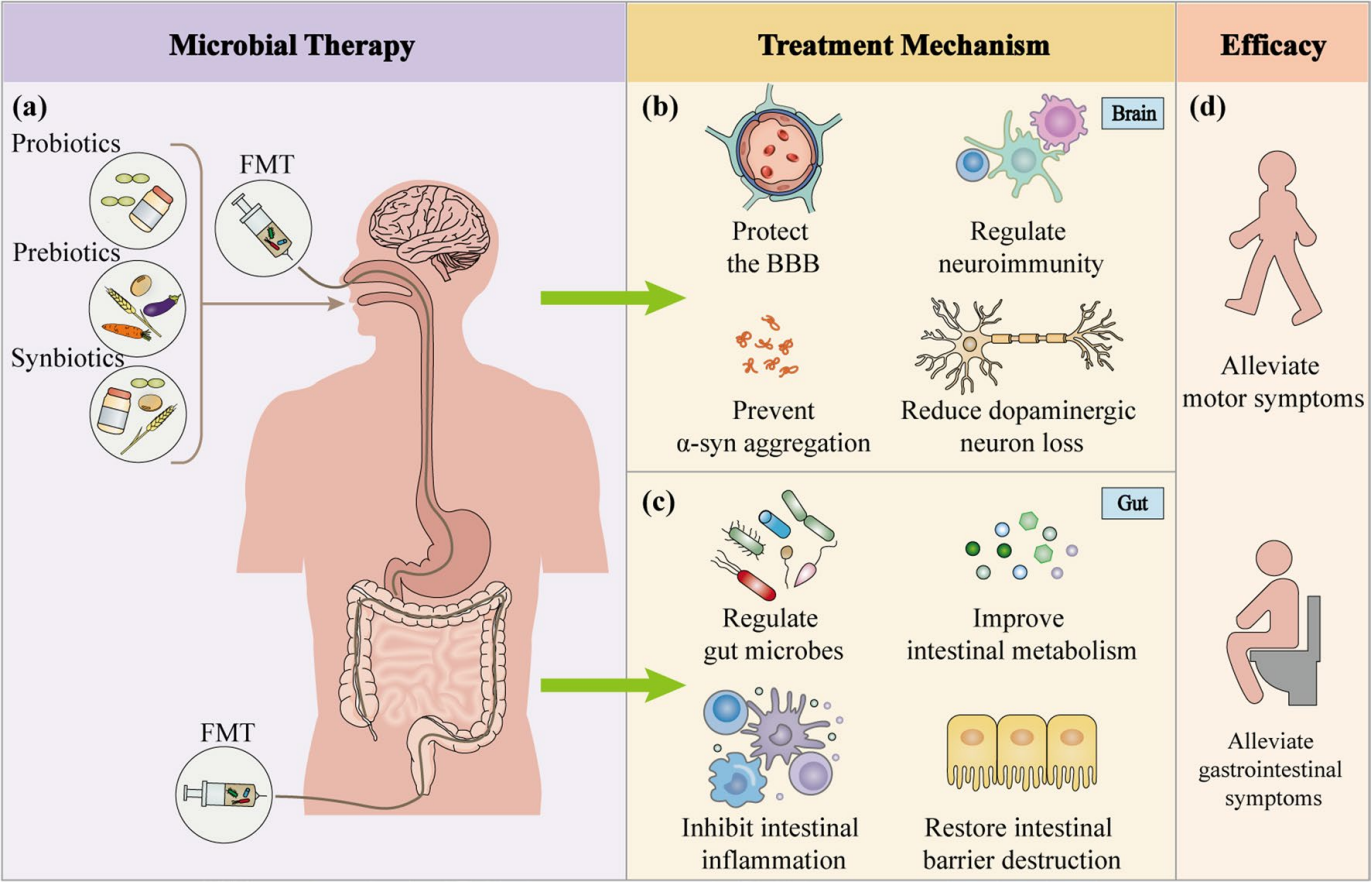
Microbial therapies for Parkinson’s disease. **a** Probiotics, prebiotics, synbiotics, and fecal microbiota transplantation are the most commonly used microbial therapies for PD. These therapies can be administered through oral, nasogastric, rectal, or colonoscopic route. **b** Microbial therapies have neuroprotective effects on the brain by reducing the blood–brain barrier damage, decreasing microglial and astrocytic activation, suppressing neuroinflammation, and inhibiting α-syn aggregation, thereby preventing the death of dopaminergic neurons. **c** In the gut, microbial therapies can regulate gut microbes, improve intestinal metabolism, modulate the intestinal mucosal immune system, inhibit gut inflammation, and restore gut barrier damage, resulting in improved intestinal symptoms. **d** In conclusion, microbial therapies relieve nonmotor symptoms of PD, particularly constipation, as well as the motor symptoms through multiple pathways

Conversion of the dysfunctional gut flora to health-related gut flora is a major principle of gut microbial therapy. With growing knowledge of gut flora and PD, scientists have investigated therapeutic strategies for PD by modifying gut microbes. Probiotics, prebiotics, synbiotics, fecal microbiome transplantation (FMT), and other microbial therapies have been shown to relieve gastrointestinal symptoms; some can even relieve motor symptoms. These microbial therapies have provided new options for the treatment of PD (Table 5, Fig. 5).

**Table 5:** Microbial therapies for PD

Ref.	Sample size	Type	Treatment duration	Main results
*Probiotics*				
[263]	Probiotics treatment: 25 Placebo: 25	*Lactobacillus acidophilus, Bifidobacterium bifidum, L. reuteri,* and *L. fermentum*	12 weeks	Downregulated the gene expression of IL-1, IL-8, and TNF-α and upregulated TGF-β and PPAR-γ in PBMC
[264]	Probiotics treatment: 30 Placebo: 30	*L. acidophilus, B. bifidum, L. reuteri,* and* L. fermentum*	12 weeks	Decreased MDS-UPDRS, hs-CRP, and malondialdehyde, enhanced glutathione, and improved insulin sensitivity
[265]	Probiotics treatment: 34 Placebo: 38	*L. acidophilus, L. reuteri, L. gasseri, L. rhamnosus, B. bifidum, B. longum, Enterococcus faecalis, E. faecium*	4 weeks	Improved constipation symptoms
[266]	Probiotics treatment: 23 Placebo: 23	*Bacillus licheniformis, L. acidophilus, B. longum, E. faecalis*	12 weeks	Improved constipation symptoms and positively affected gut microbiota
*Prebiotics*				
[267]	PD: 19	A diet rich in insoluble fiber	2 months	Improved constipation symptoms and increased plasma levodopa bioavailability and motor function
[268]	Resistant starch: 32 PD, 30 control Solely dietary instructions: 25 PD	Resistant starch	8 weeks	Improved nonmotor symptom scores, increased fecal butyrate, and decreased fecal calprotectin levels
[191]	Newly diagnosed, non-medicated PD: 10 Treated PD: 10	Prebiotic fiber	10 days	Prebiotic intervention was well tolerated and safe, associated with beneficial biological changes in microbiota, SCFA, inflammation, and neurofilament light chain, and may improve clinical symptoms (i.e., gastrointestinal symptoms and UPDRS)
*Synbiotics*				
[269]	Multiple probiotic strains and prebiotic fiber: 80 Placebo: 40	Multiple probiotic strains: S*treptococcus salivarius* subsp. *thermophilus, E. faecium, L. rhamnosus* GG, *L. acidophilus, L. plantarum, L. paracasei, L. delbrueckii* subsp. *bulgaricus,* and* Bifidobacterium* Prebiotic fiber: fructo-oligosaccharides	4 weeks	Improved constipation symptoms
[270]	Multi-strain probiotic (Hexbio®): 22 Placebo: 26	Multi-strain probiotic (*Lactobacillus* sp. and *Bifidobacterium* sp.) with fructo-oligosaccaride	8 weeks	Improved bowel opening frequency and whole gut transit time
*Fecal microbiota transplantation*				
[271]	PD: 15	Purified fecal microbiota suspension	Once	Relieved motor and nonmotor symptoms with acceptable safety
[272]	PD: 6	Fecal suspension	Once	Relieved motor and nonmotor symptoms, including constipation
[273]	PD: 11	Frozen fecal microbiota	Once	Reconstructed gut microbiota and improved motor and nonmotor symptoms
[274]	PD: 12	FMT capsules (*n* = 8) placebo (*n* = 4)	Twice weekly for 12 weeks	Improved subjective motor and non-motor complaints, intestinal microbiota diversity, gut transit, and motility

IL-1: interleukin-1, IL-8: interleukin-8, TNF-α: tumor necrosis factor alpha, TGF-β: transforming growth factor beta, PPAR-γ: peroxisome proliferator-activated receptor gamma, PBMC: peripheral blood mononuclear cell, MDS-UPDRS: Movement Disorder Society-Unified Parkinson’s Disease Rating Scale, hs-CRP: high-sensitivity C-reactive protein, PD: Parkinson’s disease, SCFA: short-chain fatty acid

### Probiotics

Probiotics are “live microorganisms that, when administered in adequate amounts, confer a health benefit on the host” [275]. Typical probiotics consist mainly of bacteria naturally produced in the human intestine, usually *Lactobacillus*, *Bifidobacterium*, *Saccharomyces*, and combinations of different beneficial bacteria [276]. Probiotics are available from food, supplements, medications, and formula [275]. Increasing evidence has shown that probiotics stimulate intestinal motility and play a protective role as they strengthen the integrity of the intestinal epithelium, prevent disruption of the intestinal barrier, promote a balanced mucosal immune system, and inhibit harmful microorganisms [277, 278].

Probiotics protect against PD; however, clinical and preclinical data supporting this finding are lacking. Probiotics relieve several motor and nonmotor symptoms, particularly the gastrointestinal symptoms. The mechanisms by which probiotics could improve PD symptoms may involve the intestinal environmental change, inhibition of harmful intestinal bacteria, decreased inflammation, prevention of antioxidant stress, and improvement of neuronutrition [243, 279–285]. A study of an established *C. elegans* synucleinopathy model showed that the probiotic *Bacillus subtilis* strain PXN21 suppresses and eliminates α-syn accumulation. The bacteria use metabolites and biofilm development to trigger host defense mechanisms, such as DAF-16/FOXO and sphingolipid metabolism [241]. Surprisingly, since some common probiotics such as *Lactobacillus* and *Bifidobacterium* have been found consistently elevated in patients with PD, the exact role of probiotics in PD is questionable.

### Prebiotics

A prebiotic is “a substrate that is selectively utilized by host microorganisms conferring a health benefit” [286]. Generally, prebiotics are regarded as non-digestible dietary components that encourage the development and activity of certain microbial genera to promote the recipient’s health [287, 288]. Unlike probiotics, prebiotics do not include living bacteria, but rather consist of dietary fiber. Prebiotics are most frequently found in foods. Some synthetic prebiotics include inulin, galactooligosaccharides, fructo-oligosaccharides, and SCFAs [289].

Prebiotics are becoming more widely used in clinical settings owing to their low risk of side effects, ease of administration, and significant impact on the composition and function of gut microbiota [290]. However, although clinical studies on the correlation between PD and prebiotics are very limited, evidence shows that prebiotics can modulate immune function, improve bowel motility and constipation, and offer other aspects of gastrointestinal health, indicating their potential clinical value [291, 292]. In MPTP-treated mice, polymannuronic acid (PM) reduced inflammation in the intestine, brain and circulation, and enhanced the intestinal barrier and BBB integrity to protect against the development of PD. PM may affect the brain-gut microbiome axis through gut microbiota-derived SCFAs [293].

### Synbiotics

Synbiotics are synergistic mixtures of probiotics and prebiotics, in which the prebiotic ingredients are selectively beneficial to the metabolism or growth of probiotics, resulting in a beneficial effect on host health [294]. The combination of the probiotics and prebiotics can be more effective than each alone [289]. Recently, the use of a new synbiotic consisting of PM and *Lacticaseibacillus rhamnosus* GG (LGG) in a PD animal model showed neuroprotective effects. The synbiotic can increase the expression of the tyrosine hydroxylase gene and/or protein, prevent the death of dopaminergic neurons, and improve motor function. The mechanisms of action include the anti-inflammatory and anti-apoptotic effects of SCFAs provided by PM, as well as the improved expression of neurotrophic factors by striatal glial cells and increased abundance of Clostridiales offered by LGG [295].

### FMT

FMT, also known as fecal transplantation or fecal bacteriotherapy, is a technique that delivers the stool from healthy donors into a patient’s gastrointestinal tract [42, 296]. This technique can comprehensively and extensively restore the abnormal gut microbiota and has been approved for clinical use by the WHO and the FDA as a treatment for gastrointestinal infections or other diseases [297]. FMT may regulate the intestinal microbiome through immune, endocrine, metabolic, and neurological mechanisms, thereby affecting the symptoms of neurological disorders. It has been proven that patients with different neurological diseases, including PD, benefit from FMT therapy [298–300].

FMT significantly attenuates intestinal microbial metabolic disorders in PD mice, reduces intestinal inflammation and barrier disruption, attenuates BBB damage, reduces nigrostriatal microglia and astrocyte activation, inhibits neuroinflammation, and reduces gut and brain TLR4/TNF-α signaling pathway components, thereby protecting dopaminergic neurons and increasing striatal dopamine and 5-HT content [142, 196, 301]. FMT improves motor and gastrointestinal dysfunction in PD, leads to healthy intestinal flora diversity in patients, and improves nonmotor symptoms, including sleep, quality of life, anxiety, and depression. A recent study with 12 weeks of continuous treatment and 9 months of follow-up demonstrated that PD FMT was well tolerated and resulted in improvements of subjective symptoms and objective intestinal markers [274].

Despite the positive results obtained after FMT, the treatment is associated with several challenges, such as ethical issues, selection of suitable donors, handling of fecal transplants, the optimal volume and frequency of transplants, risk and benefit assessment, and long-term safety [302–305]. It is hypothesized that FMT only replaces microorganisms in the intestinal lumen without changing mucosal microorganisms [302]. Notably, clinical trials on FMT treatment for PD have not shown stable long-term efficacy [271, 306].

## Conclusions and perspectives

High-throughput sequencing technology has enabled remarkable advancements in gut microbiota research. Although considerable indirect evidence implies that the microbiome may contribute to PD, conclusive evidence is lacking. It is extremely difficult to demonstrate the precise molecular pathways by which the microbiome promotes the pathogenesis of PD. Notably, recent developments in tissue culture technologies, particularly the development of human intestinal organoids and their integration with more elaborate organ-on-a-chip setups, provide excellent model systems for studying host-microbe interactions with a high degree of clinical relevance [250, 252, 307]. Organoids most faithfully recapitulate intestinal cell types, and gut-on-chips are biomimetics recapitulating intestinal physiology, allowing the detection of molecular exchanges between microbes and human cells and their effects, thus providing a reproducible and scalable platform for causal and translational gut microbiome research. Excitingly, advances in organ-on-chip technology have made it possible to utilize multiorgan-on-chip system to mimic the effects of the gut microbiome on extraintestinal organs, which provide technical support for studying CNS disorders, including PD, and for the refinement of the microbiota–gut–brain axis concept [308, 309]. It is believed that in the future, with the deepening of clinical studies, the support of mechanistic modeling approaches and improved in vitro simulation, the specific mechanism of the role of gut microorganisms in the development of PD will be revealed.

Most existing studies on gut microbes in PD are cross-sectional studies, which cannot sufficiently indicate causal relationships between gut microbes and the pathogenesis of PD. Thus, further longitudinal research, especially that of patients with new-onset, unmedicated, or even prodromal PD, is needed to advance our knowledge of the mechanisms underlying the correlation between gut microbes and PD. In addition, further expansion of research subjects, such as the inclusion of patients with atypical PD (e.g., multiple system atrophy and progressive supranuclear palsy), may help elucidate the discriminatory power of gut microbes between PD and similar disorders. Finally, evaluation of extended microbiome data using multiomics, including metagenomics, viral metagenomics, transcriptomics, proteomics, and metabolomics, may provide a multidimensional view of the mechanisms of PD.

### Supplementary Information

**Additional file 1: Table S1. **Microbiome alterations in clinical cohorts of Parkinson’s disease.

## Data Availability

Not applicable.

## Abbreviations

α-syn: Alpha-synuclein
AFMT: (S)-α-fluoromethyltyrosine
BBB: Blood–brain barrier
COMT: Catechol-O-methyl transferase
ENS: Enteric nervous system
FMT: Fecal microbiome transplantation
GFAP: Glial fibrillary acidic protein
IBD: Inflammatory bowel disease
IL: Interleukin
L-DOPA: Levodopa
LPS: Lipopolysaccharide
MDS: Movement Disorders Society
MPTP: 1-Methyl-4-phenyl-1,2,3,6-tetrahydropyridine
PD: Parkinson’s disease
PM: Polymannuronic acid
PNS: Peripheral nervous system
SCFA: Short-chain fatty acid
SN: Substantia nigra
TLRs: Toll-like receptors
TNF-α: Tumor necrosis factor alpha
tyrDC: Tyrosine decarboxylase

## References

[1] Marras C, Canning CG, Goldman SM (2019). Environment, lifestyle, and Parkinson's disease: implications for prevention in the next decade. Mov Disord.

[2] Obeso JA, Jon Stoessl A, Stamelou M (2017). Editors' note: the 200th anniversary of the shaking palsy. Mov Disord.

[3] Zundler S, Gunther C, Kremer AE, Zaiss MM, Rothhammer V, Neurath MF (2023). Gut immune cell trafficking: inter-organ communication and immune-mediated inflammation. Nat Rev Gastroenterol Hepatol.

[4] Martel J, Chang SH, Ko YF, Hwang TL, Young JD, Ojcius DM (2022). Gut barrier disruption and chronic disease. Trends Endocrinol Metab.

[5] Bowman GL, Dayon L, Kirkland R, Wojcik J, Peyratout G, Severin IC (2018). Blood-brain barrier breakdown, neuroinflammation, and cognitive decline in older adults. Alzheimers Dement.

[6] Attems J, Walker L, Jellinger KA (2015). Olfaction and aging: a mini-review. Gerontology.

[7] Reeve A, Simcox E, Turnbull D (2014). Ageing and Parkinson's disease: why is advancing age the biggest risk factor?. Ageing Res Rev.

[8] Braak H, Del Tredici K, Rub U, de Vos RA, Jansen Steur EN, Braak E (2003). Staging of brain pathology related to sporadic Parkinson's disease. Neurobiol Aging.

[9] Borghammer P, Van Den Berge N (2019). Brain-first versus gut-first Parkinson's disease: a hypothesis. J Parkinsons Dis.

[10] Borghammer P, Just MK, Horsager J, Skjaerbaek C, Raunio A, Kok EH (2022). A postmortem study suggests a revision of the dual-hit hypothesis of Parkinson's disease. NPJ Parkinsons Dis.

[11] Borghammer P (2021). The alpha-Synuclein origin and connectome model (SOC model) of Parkinson's disease: explaining motor asymmetry, non-motor phenotypes, and cognitive decline. J Parkinsons Dis.

[12] Borghammer P (2023). The brain-first vs. body-first model of Parkinson's disease with comparison to alternative models. J Neural Transm (Vienna).

[13] Borghammer P, Horsager J, Andersen K, Van Den Berge N, Raunio A, Murayama S (2021). Neuropathological evidence of body-first vs. brain-first Lewy body disease. Neurobiol Dis.

[14] Horsager J, Andersen KB, Knudsen K, Skjaerbaek C, Fedorova TD, Okkels N (2020). Brain-first versus body-first Parkinson's disease: a multimodal imaging case-control study. Brain.

[15] Knudsen K, Fedorova TD, Hansen AK, Sommerauer M, Haase AM, Svendsen KB (2019). Objective intestinal function in patients with idiopathic REM sleep behavior disorder. Parkinsonism Relat Disord.

[16] Knudsen K, Fedorova TD, Hansen AK, Sommerauer M, Otto M, Svendsen KB (2018). In-vivo staging of pathology in REM sleep behaviour disorder: a multimodality imaging case-control study. Lancet Neurol.

[17] Borghammer P, Horsager J (2021). The logic and pitfalls of Parkinson's as brain- versus body-first subtypes. Mov Disord.

[18] Abbott RD, Petrovitch H, White LR, Masaki KH, Tanner CM, Curb JD (2001). Frequency of bowel movements and the future risk of Parkinson's disease. Neurology.

[19] Engelender S, Isacson O (2017). The threshold theory for Parkinson's disease. Trends Neurosci.

[20] Weimers P, Halfvarson J, Sachs MC, Saunders-Pullman R, Ludvigsson JF, Peter I (2019). Inflammatory bowel disease and Parkinson's disease: a nationwide swedish cohort study. Inflamm Bowel Dis.

[21] Villumsen M, Aznar S, Pakkenberg B, Jess T, Brudek T (2019). Inflammatory bowel disease increases the risk of Parkinson's disease: a Danish nationwide cohort study 1977–2014. Gut.

[22] Peter I, Dubinsky M, Bressman S, Park A, Lu C, Chen N (2018). Anti-tumor necrosis factor therapy and incidence of Parkinson disease among patients with inflammatory bowel disease. JAMA Neurol.

[23] Park S, Kim J, Chun J, Han K, Soh H, Kang EA, et al. Patients with inflammatory bowel disease are at an increased risk of Parkinson's disease: a south korean nationwide population-based study. J Clin Med. 2019;8(8). 10.3390/jcm8081191 PMC6723604 31398905

[24] Lee HS, Lobbestael E, Vermeire S, Sabino J, Cleynen I (2021). Inflammatory bowel disease and Parkinson's disease: common pathophysiological links. Gut.

[25] Hui KY, Fernandez-Hernandez H, Hu J, Schaffner A, Pankratz N, Hsu NY, et al. Functional variants in the LRRK2 gene confer shared effects on risk for Crohn's disease and Parkinson's disease. Sci Transl Med. 2018;10(423). 10.1126/scitranslmed.aai7795 PMC6028002 29321258

[26] Fujioka S, Curry SE, Kennelly KD, Tacik P, Heckman MG, Tsuboi Y (2017). Occurrence of Crohn's disease with Parkinson's disease. Parkinsonism Relat Disord.

[27] Kang X, Ploner A, Pedersen NL, Bandres-Ciga S, Noyce AJ, Wirdefeldt K (2021). Tumor necrosis factor inhibition and Parkinson disease: a Mendelian randomization study. Neurology.

[28] Liu B, Fang F, Pedersen NL, Tillander A, Ludvigsson JF, Ekbom A (2017). Vagotomy and Parkinson disease: a Swedish register-based matched-cohort study. Neurology.

[29] Svensson E, Horvath-Puho E, Thomsen RW, Djurhuus JC, Pedersen L, Borghammer P (2015). Vagotomy and subsequent risk of Parkinson's disease. Ann Neurol.

[30] Tysnes OB, Kenborg L, Herlofson K, Steding-Jessen M, Horn A, Olsen JH (2015). Does vagotomy reduce the risk of Parkinson's disease?. Ann Neurol.

[31] Marras C, Lang AE, Austin PC, Lau C, Urbach DR (2016). Appendectomy in mid and later life and risk of Parkinson's disease: a population-based study. Mov Disord.

[32] Svensson E, Horvath-Puho E, Stokholm MG, Sorensen HT, Henderson VW, Borghammer P (2016). Appendectomy and risk of Parkinson's disease: a nationwide cohort study with more than 10 years of follow-up. Mov Disord.

[33] Palacios N, Hughes KC, Cereda E, Schwarzschild MA, Ascherio A (2018). Appendectomy and risk of Parkinson's disease in two large prospective cohorts of men and women. Mov Disord.

[34] Stokholm MG, Danielsen EH, Hamilton-Dutoit SJ, Borghammer P (2016). Pathological alpha-synuclein in gastrointestinal tissues from prodromal Parkinson disease patients. Ann Neurol.

[35] Kalaitzakis ME, Graeber MB, Gentleman SM, Pearce RK (2008). The dorsal motor nucleus of the vagus is not an obligatory trigger site of Parkinson's disease: a critical analysis of alpha-synuclein staging. Neuropathol Appl Neurobiol.

[36] Adler CH, Beach TG (2016). Neuropathological basis of nonmotor manifestations of Parkinson's disease. Mov Disord.

[37] Beach TG, Adler CH, Sue LI, Vedders L, Lue L, White Iii CL (2010). Multi-organ distribution of phosphorylated alpha-synuclein histopathology in subjects with Lewy body disorders. Acta Neuropathol.

[38] Forsyth CB, Shannon KM, Kordower JH, Voigt RM, Shaikh M, Jaglin JA (2011). Increased intestinal permeability correlates with sigmoid mucosa alpha-synuclein staining and endotoxin exposure markers in early Parkinson's disease. PLoS ONE.

[39] Hasegawa S, Goto S, Tsuji H, Okuno T, Asahara T, Nomoto K (2015). Intestinal dysbiosis and lowered serum lipopolysaccharide-binding protein in Parkinson's disease. PLoS ONE.

[40] Clairembault T, Leclair-Visonneau L, Coron E, Bourreille A, Le Dily S, Vavasseur F (2015). Structural alterations of the intestinal epithelial barrier in Parkinson's disease. Acta Neuropathol Commun.

[41] Li Z, Liang H, Hu Y, Lu L, Zheng C, Fan Y, et al. Gut bacterial profiles in Parkinson's disease: a systematic review. CNS Neurosci Ther. 2022. 10.1111/cns.13990 PMC9804059 36284437

[42] Wang Q, Luo Y, Ray Chaudhuri K, Reynolds R, Tan EK, Pettersson S (2021). The role of gut dysbiosis in Parkinson's disease: mechanistic insights and therapeutic options. Brain.

[43] Lorente-Picon M, Laguna A. New avenues for Parkinson's disease therapeutics: disease-modifying strategies based on the gut microbiota. Biomolecules. 2021;11(3). 10.3390/biom11030433 PMC7998286 33804226

[44] Chen SG, Stribinskis V, Rane MJ, Demuth DR, Gozal E, Roberts AM (2016). Exposure to the functional bacterial amyloid protein curli enhances alpha-synuclein aggregation in aged fischer 344 rats and Caenorhabditis elegans. Sci Rep.

[45] Sampson TR, Debelius JW, Thron T, Janssen S, Shastri GG, Ilhan ZE (2016). Gut microbiota regulate motor deficits and neuroinflammation in a model of Parkinson's disease. Cell.

[46] Klingelhoefer L, Reichmann H (2015). Pathogenesis of Parkinson disease–the gut-brain axis and environmental factors. Nat Rev Neurol.

[47] O'Donovan SM, Crowley EK, Brown JR, O'Sullivan O, O'Leary OF, Timmons S (2020). Nigral overexpression of alpha-synuclein in a rat Parkinson's disease model indicates alterations in the enteric nervous system and the gut microbiome. Neurogastroenterol Motil.

[48] Ulusoy A, Phillips RJ, Helwig M, Klinkenberg M, Powley TL, Di Monte DA (2017). Brain-to-stomach transfer of alpha-synuclein via vagal preganglionic projections. Acta Neuropathol.

[49] Tan AH, Lim SY, Lang AE (2022). The microbiome-gut-brain axis in Parkinson disease—from basic research to the clinic. Nat Rev Neurol.

[50] Leclair-Visonneau L, Neunlist M, Derkinderen P, Lebouvier T (2020). The gut in Parkinson's disease: bottom-up, top-down, or neither?. Neurogastroenterol Motil.

[51] Pellegrini C, Fornai M, Colucci R, Tirotta E, Blandini F, Levandis G (2016). Alteration of colonic excitatory tachykininergic motility and enteric inflammation following dopaminergic nigrostriatal neurodegeneration. J Neuroinflammation.

[52] Kim JS, Sung HY (2015). Gastrointestinal autonomic dysfunction in Patients with Parkinson's disease. J Mov Disord.

[53] Vizcarra JA, Wilson-Perez HE, Fasano A, Espay AJ (2018). Small intestinal bacterial overgrowth in Parkinson's disease: tribulations of a trial. Parkinsonism Relat Disord.

[54] Chen H, Zhao EJ, Zhang W, Lu Y, Liu R, Huang X (2015). Meta-analyses on prevalence of selected Parkinson's nonmotor symptoms before and after diagnosis. Transl Neurodegener.

[55] Cersosimo MG, Raina GB, Pecci C, Pellene A, Calandra CR, Gutierrez C (2013). Gastrointestinal manifestations in Parkinson's disease: prevalence and occurrence before motor symptoms. J Neurol.

[56] Adams-Carr KL, Bestwick JP, Shribman S, Lees A, Schrag A, Noyce AJ (2016). Constipation preceding Parkinson's disease: a systematic review and meta-analysis. J Neurol Neurosurg Psychiatry.

[57] Camacho M, Macleod AD, Maple-Grodem J, Evans JR, Breen DP, Cummins G (2021). Early constipation predicts faster dementia onset in Parkinson's disease. NPJ Parkinsons Dis.

[58] Berg D, Postuma RB, Adler CH, Bloem BR, Chan P, Dubois B (2015). MDS research criteria for prodromal Parkinson's disease. Mov Disord.

[59] Edwards LL, Quigley EM, Pfeiffer RF (1992). Gastrointestinal dysfunction in Parkinson's disease: frequency and pathophysiology. Neurology.

[60] Minguez-Castellanos A, Chamorro CE, Escamilla-Sevilla F, Ortega-Moreno A, Rebollo AC, Gomez-Rio M (2007). Do alpha-synuclein aggregates in autonomic plexuses predate Lewy body disorders?: a cohort study. Neurology.

[61] Wakabayashi K, Takahashi H, Takeda S, Ohama E, Ikuta F (1988). Parkinson's disease: the presence of Lewy bodies in Auerbach's and Meissner's plexuses. Acta Neuropathol.

[62] Braak H, Rub U, Gai WP, Del Tredici K (2003). Idiopathic Parkinson's disease: possible routes by which vulnerable neuronal types may be subject to neuroinvasion by an unknown pathogen. J Neural Transm (Vienna).

[63] Hawkes CH, Del Tredici K, Braak H (2007). Parkinson's disease: a dual-hit hypothesis. Neuropathol Appl Neurobiol.

[64] Tansey MG, Wallings RL, Houser MC, Herrick MK, Keating CE, Joers V. Inflammation and immune dysfunction in Parkinson disease. Nat Rev Immunol. 2022. 10.1038/s41577-022-00684-6 PMC8895080 35246670

[65] Kim S, Kwon SH, Kam TI, Panicker N, Karuppagounder SS, Lee S (2019). Transneuronal propagation of pathologic alpha-synuclein from the gut to the brain models Parkinson's disease. Neuron.

[66] Berg D, Borghammer P, Fereshtehnejad SM, Heinzel S, Horsager J, Schaeffer E (2021). Prodromal Parkinson disease subtypes—key to understanding heterogeneity. Nat Rev Neurol.

[67] Arotcarena ML, Dovero S, Prigent A, Bourdenx M, Camus S, Porras G (2020). Bidirectional gut-to-brain and brain-to-gut propagation of synucleinopathy in non-human primates. Brain.

[68] Fenyi A, Duyckaerts C, Bousset L, Braak H, Del Tredici K, Melki R, et al. Seeding propensity and characteristics of pathogenic alphasyn assemblies in formalin-fixed human tissue from the enteric nervous system, olfactory bulb, and brainstem in cases staged for Parkinson's disease. Cells. 2021;10(1). 10.3390/cells10010139 PMC7828121 33445653

[69] Li M, Wang B, Zhang M, Rantalainen M, Wang S, Zhou H (2008). Symbiotic gut microbes modulate human metabolic phenotypes. Proc Natl Acad Sci U S A.

[70] Goldsmith JR, Sartor RB (2014). The role of diet on intestinal microbiota metabolism: downstream impacts on host immune function and health, and therapeutic implications. J Gastroenterol.

[71] Abt MC, Artis D (2009). The intestinal microbiota in health and disease: the influence of microbial products on immune cell homeostasis. Curr Opin Gastroenterol.

[72] Nicholson JK, Holmes E, Kinross J, Burcelin R, Gibson G, Jia W (2012). Host-gut microbiota metabolic interactions. Science.

[73] Holmes E, Li JV, Marchesi JR, Nicholson JK (2012). Gut microbiota composition and activity in relation to host metabolic phenotype and disease risk. Cell Metab.

[74] Sommer F, Backhed F (2013). The gut microbiota–masters of host development and physiology. Nat Rev Microbiol.

[75] Lynch SV, Pedersen O (2016). The human intestinal microbiome in health and disease. N Engl J Med.

[76] Morais LH, Schreiber HL, Mazmanian SK (2021). The gut microbiota-brain axis in behaviour and brain disorders. Nat Rev Microbiol.

[77] Rhee SH, Pothoulakis C, Mayer EA (2009). Principles and clinical implications of the brain-gut-enteric microbiota axis. Nat Rev Gastroenterol Hepatol.

[78] Montanari M, Imbriani P, Bonsi P, Martella G, Peppe A. Beyond the microbiota: understanding the role of the enteric nervous system in Parkinson's disease from mice to human. Biomedicines. 2023;11(6). 10.3390/biomedicines11061560 PMC10295288 37371655

[79] Chalazonitis A, Rao M (2018). Enteric nervous system manifestations of neurodegenerative disease. Brain Res.

[80] Chalazonitis A, Rao M, Sulzer D (2022). Similarities and differences between nigral and enteric dopaminergic neurons unravel distinctive involvement in Parkinson's disease. NPJ Parkinsons Dis.

[81] Giancola F, Torresan F, Repossi R, Bianco F, Latorre R, Ioannou A, et al. Downregulation of neuronal vasoactive intestinal polypeptide in Parkinson's disease and chronic constipation. Neurogastroenterol Motil. 2017;29(5). 10.1111/nmo.12995 PMC5393951 27891695

[82] Natale G, Ryskalin L, Morucci G, Lazzeri G, Frati A, Fornai F. The baseline structure of the enteric nervous system and its role in Parkinson's disease. Life (Basel). 2021;11(8). 10.3390/life11080732 PMC8400095 34440476

[83] Gries M, Christmann A, Schulte S, Weyland M, Rommel S, Martin M (2021). Parkinson mice show functional and molecular changes in the gut long before motoric disease onset. Mol Neurodegener.

[84] Wang L, Magen I, Yuan PQ, Subramaniam SR, Richter F, Chesselet MF (2012). Mice overexpressing wild-type human alpha-synuclein display alterations in colonic myenteric ganglia and defecation. Neurogastroenterol Motil.

[85] Santos SF, de Oliveira HL, Yamada ES, Neves BC, Pereira A (2019). The gut and Parkinson's disease-a bidirectional pathway. Front Neurol.

[86] Scheperjans F, Aho V, Pereira PA, Koskinen K, Paulin L, Pekkonen E (2015). Gut microbiota are related to Parkinson's disease and clinical phenotype. Mov Disord.

[87] Bedarf JR, Hildebrand F, Coelho LP, Sunagawa S, Bahram M, Goeser F (2017). Functional implications of microbial and viral gut metagenome changes in early stage L-DOPA-naive Parkinson's disease patients. Genome Med.

[88] Li W, Wu X, Hu X, Wang T, Liang S, Duan Y (2017). Structural changes of gut microbiota in Parkinson's disease and its correlation with clinical features. Sci China Life Sci.

[89] Heintz-Buschart A, Pandey U, Wicke T, Sixel-Doring F, Janzen A, Sittig-Wiegand E (2018). The nasal and gut microbiome in Parkinson's disease and idiopathic rapid eye movement sleep behavior disorder. Mov Disord.

[90] Qian Y, Yang X, Xu S, Wu C, Song Y, Qin N (2018). Alteration of the fecal microbiota in Chinese patients with Parkinson's disease. Brain Behav Immun.

[91] Aho VTE, Pereira PAB, Voutilainen S, Paulin L, Pekkonen E, Auvinen P (2019). Gut microbiota in Parkinson's disease: temporal stability and relations to disease progression. EBioMedicine.

[92] Barichella M, Severgnini M, Cilia R, Cassani E, Bolliri C, Caronni S (2019). Unraveling gut microbiota in Parkinson's disease and atypical parkinsonism. Mov Disord.

[93] Cirstea MS, Yu AC, Golz E, Sundvick K, Kliger D, Radisavljevic N (2020). Microbiota composition and metabolism are associated with gut function in Parkinson's disease. Mov Disord.

[94] Qian Y, Yang X, Xu S, Huang P, Li B, Du J (2020). Gut metagenomics-derived genes as potential biomarkers of Parkinson's disease. Brain.

[95] Rosario D, Bidkhori G, Lee S, Bedarf J, Hildebrand F, Le Chatelier E (2021). Systematic analysis of gut microbiome reveals the role of bacterial folate and homocysteine metabolism in Parkinson's disease. Cell Rep.

[96] Tan AH, Chong CW, Lim SY, Yap IKS, Teh CSJ, Loke MF (2021). Gut microbial ecosystem in parkinson disease: new clinicobiological insights from multi-omics. Ann Neurol.

[97] Wallen ZD, Demirkan A, Twa G, Cohen G, Dean MN, Standaert DG (2022). Metagenomics of Parkinson's disease implicates the gut microbiome in multiple disease mechanisms. Nat Commun.

[98] Zhang K, Paul KC, Jacobs JP, Chou HL, Duarte Folle A, Del Rosario I (2022). Parkinson's disease and the gut microbiome in rural California. J Parkinsons Dis.

[99] Li C, Cui L, Yang Y, Miao J, Zhao X, Zhang J (2019). Gut microbiota differs between Parkinson's disease patients and healthy controls in Northeast China. Front Mol Neurosci.

[100] Keshavarzian A, Green SJ, Engen PA, Voigt RM, Naqib A, Forsyth CB (2015). Colonic bacterial composition in Parkinson's disease. Mov Disord.

[101] Unger MM, Spiegel J, Dillmann KU, Grundmann D, Philippeit H, Burmann J (2016). Short chain fatty acids and gut microbiota differ between patients with Parkinson's disease and age-matched controls. Parkinsonism Relat Disord.

[102] Hill-Burns EM, Debelius JW, Morton JT, Wissemann WT, Lewis MR, Wallen ZD (2017). Parkinson's disease and Parkinson's disease medications have distinct signatures of the gut microbiome. Mov Disord.

[103] Hopfner F, Kunstner A, Muller SH, Kunzel S, Zeuner KE, Margraf NG (2017). Gut microbiota in Parkinson disease in a northern German cohort. Brain Res.

[104] Huang B, Chau SWH, Liu Y, Chan JWY, Wang J, Ma SL (2023). Gut microbiome dysbiosis across early Parkinson's disease, REM sleep behavior disorder and their first-degree relatives. Nat Commun.

[105] Nishiwaki H, Hamaguchi T, Ito M, Ishida T, Maeda T, Kashihara K, et al. Short-chain fatty acid-producing gut microbiota is decreased in Parkinson's disease but not in rapid-eye-movement sleep behavior disorder. mSystems. 2020;5(6). 10.1128/mSystems.00797-20 PMC7771407 33293403

[106] Duvallet C, Gibbons SM, Gurry T, Irizarry RA, Alm EJ (2017). Meta-analysis of gut microbiome studies identifies disease-specific and shared responses. Nat Commun.

[107] Lin A, Zheng W, He Y, Tang W, Wei X, He R (2018). Gut microbiota in patients with Parkinson's disease in southern China. Parkinsonism Relat Disord.

[108] Pietrucci D, Cerroni R, Unida V, Farcomeni A, Pierantozzi M, Mercuri NB (2019). Dysbiosis of gut microbiota in a selected population of Parkinson's patients. Parkinsonism Relat Disord.

[109] Lin CH, Chen CC, Chiang HL, Liou JM, Chang CM, Lu TP (2019). Altered gut microbiota and inflammatory cytokine responses in patients with Parkinson's disease. J Neuroinflammation.

[110] Lebouvier T, Neunlist M, Bruleydes Varannes S, Coron E, Drouard A, N’Guyen JM (2010). Colonic biopsies to assess the neuropathology of Parkinson's disease and its relationship with symptoms. PLoS ONE.

[111] Marras C, Lang A (2013). Parkinson's disease subtypes: lost in translation?. J Neurol Neurosurg Psychiatry.

[112] van Rooden SM, Colas F, Martinez-Martin P, Visser M, Verbaan D, Marinus J (2011). Clinical subtypes of Parkinson's disease. Mov Disord.

[113] Minato T, Maeda T, Fujisawa Y, Tsuji H, Nomoto K, Ohno K (2017). Progression of Parkinson's disease is associated with gut dysbiosis: two-year follow-up study. PLoS ONE.

[114] Jones JD, Rahmani E, Garcia E, Jacobs JP (2020). Gastrointestinal symptoms are predictive of trajectories of cognitive functioning in de novo Parkinson's disease. Parkinsonism Relat Disord.

[115] He Y, Wu W, Zheng HM, Li P, McDonald D, Sheng HF (2018). Regional variation limits applications of healthy gut microbiome reference ranges and disease models. Nat Med.

[116] Groussin M, Poyet M, Sistiaga A, Kearney SM, Moniz K, Noel M (2021). Elevated rates of horizontal gene transfer in the industrialized human microbiome. Cell.

[117] Salim S, Ahmad F, Banu A, Mohammad F (2023). Gut microbiome and Parkinson's disease: perspective on pathogenesis and treatment. J Adv Res.

[118] Elfil M, Kamel S, Kandil M, Koo BB, Schaefer SM (2020). Implications of the gut microbiome in Parkinson's disease. Mov Disord.

[119] Aho VTE, Houser MC, Pereira PAB, Chang J, Rudi K, Paulin L (2021). Relationships of gut microbiota, short-chain fatty acids, inflammation, and the gut barrier in Parkinson's disease. Mol Neurodegener.

[120] Friedland RP (2015). Mechanisms of molecular mimicry involving the microbiota in neurodegeneration. J Alzheimers Dis.

[121] Wang C, Lau CY, Ma F, Zheng C. Genome-wide screen identifies curli amyloid fibril as a bacterial component promoting host neurodegeneration. Proc Natl Acad Sci USA. 2021;118(34). 10.1073/pnas.2106504118 PMC8403922 34413194

[122] Schwartz K, Boles BR (2013). Microbial amyloids–functions and interactions within the host. Curr Opin Microbiol.

[123] Oli MW, Otoo HN, Crowley PJ, Heim KP, Nascimento MM, Ramsook CB (2012). Functional amyloid formation by Streptococcus mutans. Microbiology (Reading).

[124] Taglialegna A, Lasa I, Valle J (2016). Amyloid structures as biofilm matrix scaffolds. J Bacteriol.

[125] Sampson TR, Challis C, Jain N, Moiseyenko A, Ladinsky MS, Shastri GG, et al. A gut bacterial amyloid promotes alpha-synuclein aggregation and motor impairment in mice. Elife. 2020;9. 10.7554/eLife.53111 PMC7012599 32043464

[126] Lai F, Jiang R, Xie W, Liu X, Tang Y, Xiao H (2018). Intestinal pathology and gut microbiota alterations in a methyl-4-phenyl-1,2,3,6-tetrahydropyridine (MPTP) mouse model of Parkinson's disease. Neurochem Res.

[127] Bloem BR, Okun MS, Klein C (2021). Parkinson's disease. Lancet.

[128] Xanthos DN, Sandkuhler J (2014). Neurogenic neuroinflammation: inflammatory CNS reactions in response to neuronal activity. Nat Rev Neurosci.

[129] Garcia-Esparcia P, Llorens F, Carmona M, Ferrer I (2014). Complex deregulation and expression of cytokines and mediators of the immune response in Parkinson's disease brain is region dependent. Brain Pathol.

[130] Hirsch EC, Hunot S (2009). Neuroinflammation in Parkinson's disease: a target for neuroprotection?. Lancet Neurol.

[131] Franceschi C, Garagnani P, Parini P, Giuliani C, Santoro A (2018). Inflammaging: a new immune-metabolic viewpoint for age-related diseases. Nat Rev Endocrinol.

[132] Perez-Pardo P, Dodiya HB, Engen PA, Forsyth CB, Huschens AM, Shaikh M (2019). Role of TLR4 in the gut-brain axis in Parkinson's disease: a translational study from men to mice. Gut.

[133] Devos D, Lebouvier T, Lardeux B, Biraud M, Rouaud T, Pouclet H (2013). Colonic inflammation in Parkinson's disease. Neurobiol Dis.

[134] Houser MC, Chang J, Factor SA, Molho ES, Zabetian CP, Hill-Burns EM (2018). Stool immune profiles evince gastrointestinal inflammation in Parkinson's disease. Mov Disord.

[135] Mulak A, Koszewicz M, Panek-Jeziorna M, Koziorowska-Gawron E, Budrewicz S (2019). Fecal calprotectin as a marker of the gut immune system activation is elevated in Parkinson's disease. Front Neurosci.

[136] Schwiertz A, Spiegel J, Dillmann U, Grundmann D, Burmann J, Fassbender K (2018). Fecal markers of intestinal inflammation and intestinal permeability are elevated in Parkinson's disease. Parkinsonism Relat Disord.

[137] Campos-Acuna J, Elgueta D, Pacheco R (2019). T-cell-driven inflammation as a mediator of the gut-brain axis involved in Parkinson's disease. Front Immunol.

[138] Erny D, Dokalis N, Mezo C, Castoldi A, Mossad O, Staszewski O (2021). Microbiota-derived acetate enables the metabolic fitness of the brain innate immune system during health and disease. Cell Metab.

[139] Wang X, Sun G, Feng T, Zhang J, Huang X, Wang T (2019). Sodium oligomannate therapeutically remodels gut microbiota and suppresses gut bacterial amino acids-shaped neuroinflammation to inhibit Alzheimer's disease progression. Cell Res.

[140] de Theije CG, Wopereis H, Ramadan M, van Eijndthoven T, Lambert J, Knol J (2014). Altered gut microbiota and activity in a murine model of autism spectrum disorders. Brain Behav Immun.

[141] Sun MF, Shen YQ (2018). Dysbiosis of gut microbiota and microbial metabolites in Parkinson's disease. Ageing Res Rev.

[142] Zhao Z, Ning J, Bao XQ, Shang M, Ma J, Li G (2021). Fecal microbiota transplantation protects rotenone-induced Parkinson's disease mice via suppressing inflammation mediated by the lipopolysaccharide-TLR4 signaling pathway through the microbiota-gut-brain axis. Microbiome.

[143] Elahy M, Jackaman C, Mamo JC, Lam V, Dhaliwal SS, Giles C (2015). Blood-brain barrier dysfunction developed during normal aging is associated with inflammation and loss of tight junctions but not with leukocyte recruitment. Immun Ageing.

[144] Gray MT, Woulfe JM (2015). Striatal blood-brain barrier permeability in Parkinson's disease. J Cereb Blood Flow Metab.

[145] Matheoud D, Cannon T, Voisin A, Penttinen AM, Ramet L, Fahmy AM (2019). Intestinal infection triggers Parkinson's disease-like symptoms in Pink1(-/-) mice. Nature.

[146] Schirmer M, Garner A, Vlamakis H, Xavier RJ (2019). Microbial genes and pathways in inflammatory bowel disease. Nat Rev Microbiol.

[147] Fang P, Kazmi SA, Jameson KG, Hsiao EY (2020). The microbiome as a modifier of neurodegenerative disease risk. Cell Host Microbe.

[148] Tsafaras G, Baekelandt V (2022). The role of LRRK2 in the periphery: link with Parkinson's disease and inflammatory diseases. Neurobiol Dis.

[149] Cabezudo D, Baekelandt V, Lobbestael E (2020). Multiple-hit hypothesis in Parkinson's disease: LRRK2 and inflammation. Front Neurosci.

[150] Derkinderen P, de Guilhem de Lataillade A, Neunlist M, Rolli-Derkinderen M. Mild Chronic colitis triggers parkinsonism in LRRK2 mutant mice through activating TNF-alpha pathway. Mov Disord. 2022;37(3):664–65. 10.1002/mds.28948 35100471

[151] Cabezudo D, Tsafaras G, Van Acker E, Van den Haute C, Baekelandt V (2023). Mutant LRRK2 exacerbates immune response and neurodegeneration in a chronic model of experimental colitis. Acta Neuropathol.

[152] Liao PH, Chiang HL, Shun CT, Hang JF, Chiu HM, Wu MS (2021). Colonic leucine-rich repeat kinase 2 expression is increased and associated with disease severity in patients with Parkinson's disease. Front Aging Neurosci.

[153] Nalls MA, Blauwendraat C, Vallerga CL, Heilbron K, Bandres-Ciga S, Chang D (2019). Identification of novel risk loci, causal insights, and heritable risk for Parkinson's disease: a meta-analysis of genome-wide association studies. The Lancet Neurology.

[154] de Lange KM, Moutsianas L, Lee JC, Lamb CA, Luo Y, Kennedy NA (2017). Genome-wide association study implicates immune activation of multiple integrin genes in inflammatory bowel disease. Nat Genet.

[155] Bialecka M, Kurzawski M, Klodowska-Duda G, Opala G, Juzwiak S, Kurzawski G (2007). CARD15 variants in patients with sporadic Parkinson's disease. Neurosci Res.

[156] Witoelar A, Jansen IE, Wang Y, Desikan RS, Gibbs JR, Blauwendraat C (2017). Genome-wide pleiotropy between Parkinson disease and autoimmune diseases. JAMA Neurol.

[157] Kouli A, Horne CB, Williams-Gray CH (2019). Toll-like receptors and their therapeutic potential in Parkinson's disease and alpha-synucleinopathies. Brain Behav Immun.

[158] Drouin-Ouellet J, St-Amour I, Saint-Pierre M, Lamontagne-Proulx J, Kriz J, Barker RA, et al. Toll-like receptor expression in the blood and brain of patients and a mouse model of Parkinson's disease. Int J Neuropsychopharmacol. 2014;18(6). 10.1093/ijnp/pyu103 PMC4438545 25522431

[159] Gorecki AM, Anyaegbu CC, Anderton RS (2021). TLR2 and TLR4 in Parkinson's disease pathogenesis: the environment takes a toll on the gut. Transl Neurodegener.

[160] Tursi SA, Tukel C (2018). Curli-containing enteric biofilms inside and out: matrix composition, immune recognition, and disease implications. Microbiol Mol Biol Rev.

[161] Cheng Y, Tong Q, Yuan Y, Song X, Jiang W, Wang Y (2023). alpha-Synuclein induces prodromal symptoms of Parkinson's disease via activating TLR2/MyD88/NF-kappaB pathway in Schwann cells of vagus nerve in a rat model. J Neuroinflammation.

[162] Dzamko N, Gysbers A, Perera G, Bahar A, Shankar A, Gao J (2017). Toll-like receptor 2 is increased in neurons in Parkinson's disease brain and may contribute to alpha-synuclein pathology. Acta Neuropathol.

[163] Kim C, Kwon S, Iba M, Spencer B, Rockenstein E, Mante M (2021). Effects of innate immune receptor stimulation on extracellular alpha-synuclein uptake and degradation by brain resident cells. Exp Mol Med.

[164] Lucas K, Maes M (2013). Role of the Toll Like receptor (TLR) radical cycle in chronic inflammation: possible treatments targeting the TLR4 pathway. Mol Neurobiol.

[165] Tu HY, Yuan BS, Hou XO, Zhang XJ, Pei CS, Ma YT (2021). alpha-synuclein suppresses microglial autophagy and promotes neurodegeneration in a mouse model of Parkinson's disease. Aging Cell.

[166] Fellner L, Irschick R, Schanda K, Reindl M, Klimaschewski L, Poewe W (2013). Toll-like receptor 4 is required for alpha-synuclein dependent activation of microglia and astroglia. Glia.

[167] Zhao Z, Li F, Ning J, Peng R, Shang J, Liu H (2021). Novel compound FLZ alleviates rotenone-induced PD mouse model by suppressing TLR4/MyD88/NF-kappaB pathway through microbiota-gut-brain axis. Acta Pharm Sin B.

[168] Zhao Z, Wang Y, Zhou R, Li Y, Gao Y, Tu D (2020). A novel role of NLRP3-generated IL-1beta in the acute-chronic transition of peripheral lipopolysaccharide-elicited neuroinflammation: implications for sepsis-associated neurodegeneration. J Neuroinflammation.

[169] Pellegrini C, Antonioli L, Calderone V, Colucci R, Fornai M, Blandizzi C (2020). Microbiota-gut-brain axis in health and disease: Is NLRP3 inflammasome at the crossroads of microbiota-gut-brain communications?. Prog Neurobiol.

[170] Rota L, Pellegrini C, Benvenuti L, Antonioli L, Fornai M, Blandizzi C (2019). Constipation, deficit in colon contractions and alpha-synuclein inclusions within the colon precede motor abnormalities and neurodegeneration in the central nervous system in a mouse model of alpha-synucleinopathy. Transl Neurodegener.

[171] Choi JG, Kim N, Ju IG, Eo H, Lim SM, Jang SE (2018). Oral administration of Proteus mirabilis damages dopaminergic neurons and motor functions in mice. Sci Rep.

[172] George S, Rey NL, Tyson T, Esquibel C, Meyerdirk L, Schulz E (2019). Microglia affect alpha-synuclein cell-to-cell transfer in a mouse model of Parkinson's disease. Mol Neurodegener.

[173] Bhattacharyya D, Mohite GM, Krishnamoorthy J, Gayen N, Mehra S, Navalkar A (2019). Lipopolysaccharide from gut microbiota modulates alpha-synuclein aggregation and alters its biological function. ACS Chem Neurosci.

[174] Gong Y, Chen A, Zhang G, Shen Q, Zou L, Li J (2023). Cracking brain diseases from gut microbes-mediated metabolites for precise treatment. Int J Biol Sci.

[175] Konjevod M, Nikolac Perkovic M, Saiz J, Svob Strac D, Barbas C, Rojo D (2021). Metabolomics analysis of microbiota-gut-brain axis in neurodegenerative and psychiatric diseases. J Pharm Biomed Anal.

[176] Zacharias HU, Kaleta C, Cossais F, Schaeffer E, Berndt H, Best L, et al. Microbiome and metabolome insights into the role of the gastrointestinal-brain axis in Parkinson's and Alzheimer's disease: unveiling potential therapeutic targets. Metabolites. 2022;12(12). 10.3390/metabo12121222 PMC9786685 36557259

[177] Baert F, Matthys C, Maselyne J, Van Poucke C, Van Coillie E, Bergmans B (2021). Parkinson's disease patients' short chain fatty acids production capacity after in vitro fecal fiber fermentation. NPJ Parkinsons Dis.

[178] Chen G, Ran X, Li B, Li Y, He D, Huang B (2018). Sodium butyrate inhibits inflammation and maintains epithelium barrier integrity in a TNBS-induced inflammatory bowel disease mice model. EBioMedicine.

[179] Hou Y, Li X, Liu C, Zhang M, Zhang X, Ge S (2021). Neuroprotective effects of short-chain fatty acids in MPTP induced mice model of Parkinson's disease. Exp Gerontol.

[180] Tieu K, Perier C, Caspersen C, Teismann P, Wu DC, Yan SD (2003). D-beta-hydroxybutyrate rescues mitochondrial respiration and mitigates features of Parkinson disease. J Clin Invest.

[181] Gardian G, Yang L, Cleren C, Calingasan NY, Klivenyi P, Beal MF (2004). Neuroprotective effects of phenylbutyrate against MPTP neurotoxicity. Neuromolecular Med.

[182] St Laurent R, O'Brien LM, Ahmad ST (2013). Sodium butyrate improves locomotor impairment and early mortality in a rotenone-induced Drosophila model of Parkinson's disease. Neuroscience.

[183] Ganapathy V, Thangaraju M, Prasad PD, Martin PM, Singh N (2013). Transporters and receptors for short-chain fatty acids as the molecular link between colonic bacteria and the host. Curr Opin Pharmacol.

[184] Singh N, Gurav A, Sivaprakasam S, Brady E, Padia R, Shi H (2014). Activation of Gpr109a, receptor for niacin and the commensal metabolite butyrate, suppresses colonic inflammation and carcinogenesis. Immunity.

[185] Soret R, Chevalier J, De Coppet P, Poupeau G, Derkinderen P, Segain JP (2010). Short-chain fatty acids regulate the enteric neurons and control gastrointestinal motility in rats. Gastroenterology.

[186] Clairembault T, Leclair-Visonneau L, Neunlist M, Derkinderen P (2015). Enteric glial cells: new players in Parkinson's disease?. Mov Disord.

[187] Qiao CM, Sun MF, Jia XB, Shi Y, Zhang BP, Zhou ZL (2020). Sodium butyrate causes alpha-synuclein degradation by an Atg5-dependent and PI3K/Akt/mTOR-related autophagy pathway. Exp Cell Res.

[188] Roberfroid M, Gibson GR, Hoyles L, McCartney AL, Rastall R, Rowland I (2010). Prebiotic effects: metabolic and health benefits. Br J Nutr.

[189] Cantu-Jungles TM, Rasmussen HE, Hamaker BR (2019). Potential of prebiotic butyrogenic fibers in Parkinson's disease. Front Neurol.

[190] Zhou W, Bercury K, Cummiskey J, Luong N, Lebin J, Freed CR (2011). Phenylbutyrate up-regulates the DJ-1 protein and protects neurons in cell culture and in animal models of Parkinson disease. J Biol Chem.

[191] Hall DA, Voigt RM, Cantu-Jungles TM, Hamaker B, Engen PA, Shaikh M (2023). An open label, non-randomized study assessing a prebiotic fiber intervention in a small cohort of Parkinson's disease participants. Nat Commun.

[192] Abdel-Haq R, Schlachetzki JCM, Boktor JC, Cantu-Jungles TM, Thron T, Zhang M, et al. A prebiotic diet modulates microglial states and motor deficits in alpha-synuclein overexpressing mice. Elife. 2022;11. 10.7554/eLife.81453 PMC9668333 36346385

[193] Shin C, Lim Y, Lim H, Ahn TB (2020). Plasma short-chain fatty acids in patients with Parkinson's disease. Mov Disord.

[194] Vascellari S, Palmas V, Melis M, Pisanu S, Cusano R, Uva P, et al. Gut microbiota and metabolome alterations associated with Parkinson's disease. mSystems. 2020;5(5). 10.1128/mSystems.00561-20 PMC7498685 32934117

[195] Yang X, Ai P, He X, Mo C, Zhang Y, Xu S (2022). Parkinson's Disease Is Associated with Impaired Gut-Blood Barrier for Short-Chain Fatty Acids. Mov Disord.

[196] Sun MF, Zhu YL, Zhou ZL, Jia XB, Xu YD, Yang Q (2018). Neuroprotective effects of fecal microbiota transplantation on MPTP-induced Parkinson's disease mice: gut microbiota, glial reaction and TLR4/TNF-alpha signaling pathway. Brain Behav Immun.

[197] Duan WX, Wang F, Liu JY, Liu CF. Relationship between short-chain fatty acids and Parkinson's disease: a review from pathology to clinic. Neurosci Bull. 2023. 10.1007/s12264-023-01123-9 PMC11003953 37755674

[198] Tran SM, Mohajeri MH. The role of gut bacterial metabolites in brain development, aging and disease. Nutrients. 2021;13(3). 10.3390/nu13030732 PMC7996516 33669008

[199] Colombo AV, Sadler RK, Llovera G, Singh V, Roth S, Heindl S, et al. Microbiota-derived short chain fatty acids modulate microglia and promote Abeta plaque deposition. Elife. 2021;10. 10.7554/eLife.59826 PMC8043748 33845942

[200] Chen C, Liao J, Xia Y, Liu X, Jones R, Haran J (2022). Gut microbiota regulate Alzheimer's disease pathologies and cognitive disorders via PUFA-associated neuroinflammation. Gut.

[201] Trapecar M, Wogram E, Svoboda D, Communal C, Omer A, Lungjangwa T, et al. Human physiomimetic model integrating microphysiological systems of the gut, liver, and brain for studies of neurodegenerative diseases. Sci Adv. 2021;7(5). 10.1126/sciadv.abd1707 PMC7846169 33514545

[202] Qiao CM, Sun MF, Jia XB, Li Y, Zhang BP, Zhao LP (2020). Sodium butyrate exacerbates Parkinson's disease by aggravating neuroinflammation and colonic inflammation in MPTP-induced mice model. Neurochem Res.

[203] Park J, Wang Q, Wu Q, Mao-Draayer Y, Kim CH (2019). Bidirectional regulatory potentials of short-chain fatty acids and their G-protein-coupled receptors in autoimmune neuroinflammation. Sci Rep.

[204] Mulak A, Bonaz B (2015). Brain-gut-microbiota axis in Parkinson's disease. World J Gastroenterol.

[205] Bi M, Feng L, He J, Liu C, Wang Y, Jiang H (2022). Emerging insights between gut microbiome dysbiosis and Parkinson's disease: pathogenic and clinical relevance. Ageing Res Rev.

[206] Ren T, Gao Y, Qiu Y, Jiang S, Zhang Q, Zhang J (2020). Gut microbiota altered in mild cognitive impairment compared with normal cognition in sporadic Parkinson's disease. Front Neurol.

[207] Janeiro MH, Ramirez MJ, Milagro FI, Martinez JA, Solas M. Implication of trimethylamine N-oxide (TMAO) in disease: potential biomarker or new therapeutic target. Nutrients. 2018;10(10). 10.3390/nu10101398 PMC6213249 30275434

[208] Voigt RM, Wang Z, Brown JM, Engen PA, Naqib A, Goetz CG (2022). Gut microbial metabolites in Parkinson's disease: association with lifestyle, disease characteristics, and treatment status. Neurobiol Dis.

[209] Li D, Ke Y, Zhan R, Liu C, Zhao M, Zeng A (2018). Trimethylamine-N-oxide promotes brain aging and cognitive impairment in mice. Aging Cell.

[210] Chung SJ, Rim JH, Ji D, Lee S, Yoo HS, Jung JH (2021). Gut microbiota-derived metabolite trimethylamine N-oxide as a biomarker in early Parkinson's disease. Nutrition.

[211] Chen SJ, Kuo CH, Kuo HC, Chen CC, Wu WK, Liou JM (2020). The gut metabolite trimethylamine n-oxide is associated with parkinson's disease severity and progression. Mov Disord.

[212] Sankowski B, Ksiezarczyk K, Rackowska E, Szlufik S, Koziorowski D, Giebultowicz J (2020). Higher cerebrospinal fluid to plasma ratio of p-cresol sulfate and indoxyl sulfate in patients with Parkinson's disease. Clin Chim Acta.

[213] Fasano A, Visanji NP, Liu LW, Lang AE, Pfeiffer RF (2015). Gastrointestinal dysfunction in Parkinson's disease. Lancet Neurol.

[214] Zhang Y, He X, Qian Y, Xu S, Mo C, Yan Z (2022). Plasma branched-chain and aromatic amino acids correlate with the gut microbiota and severity of Parkinson's disease. NPJ Parkinsons Dis.

[215] Tarlungeanu DC, Deliu E, Dotter CP, Kara M, Janiesch PC, Scalise M (2016). Impaired amino acid transport at the blood brain barrier is a cause of autism spectrum disorder. Cell.

[216] Agus A, Planchais J, Sokol H (2018). Gut microbiota regulation of tryptophan metabolism in health and disease. Cell Host Microbe.

[217] Chang KH, Cheng ML, Tang HY, Huang CY, Wu YR, Chen CM (2018). Alternations of metabolic profile and kynurenine metabolism in the plasma of Parkinson's disease. Mol Neurobiol.

[218] Hirayama M, Tsunoda M, Yamamoto M, Tsuda T, Ohno K (2016). Serum tyrosine-to-phenylalanine ratio is low in Parkinson's disease. J Parkinsons Dis.

[219] Nagesh Babu G, Gupta M, Paliwal VK, Singh S, Chatterji T, Roy R (2018). Serum metabolomics study in a group of Parkinson's disease patients from northern India. Clin Chim Acta.

[220] Iwasaki Y, Ikeda K, Shiojima T, Kinoshita M (1992). Increased plasma concentrations of aspartate, glutamate and glycine in Parkinson's disease. Neurosci Lett.

[221] Yan Z, Yang F, Sun L, Yu J, Sun L, Si Y (2022). Role of gut microbiota-derived branched-chain amino acids in the pathogenesis of Parkinson's disease: an animal study. Brain Behav Immun.

[222] Wang Y, Chen S, Tan J, Gao Y, Yan H, Liu Y (2021). Tryptophan in the diet ameliorates motor deficits in a rotenone-induced rat Parkinson's disease model via activating the aromatic hydrocarbon receptor pathway. Brain Behav.

[223] Leeuwendaal NK, Cryan JF, Schellekens H (2021). Gut peptides and the microbiome: focus on ghrelin. Curr Opin Endocrinol Diabetes Obes.

[224] Schellekens H, Finger BC, Dinan TG, Cryan JF (2012). Ghrelin signalling and obesity: at the interface of stress, mood and food reward. Pharmacol Ther.

[225] Andrews ZB, Erion D, Beiler R, Liu ZW, Abizaid A, Zigman J (2009). Ghrelin promotes and protects nigrostriatal dopamine function via a UCP2-dependent mitochondrial mechanism. J Neurosci.

[226] Moon M, Kim HG, Hwang L, Seo JH, Kim S, Hwang S (2009). Neuroprotective effect of ghrelin in the 1-methyl-4-phenyl-1,2,3,6-tetrahydropyridine mouse model of Parkinson's disease by blocking microglial activation. Neurotox Res.

[227] Rees D, Johnson AL, Lelos M, Smith G, Roberts LD, Phelps L, et al. Acyl-ghrelin attenuates neurochemical and motor deficits in the 6-OHDA model of Parkinson’s disease bioRxiv. 2022. 10.1007/s10571-022-01282-9 PMC10287784 36107359

[228] He X, Yuan W, Liu F, Feng J, Guo Y (2020). Acylated ghrelin is protective against 6-OHDA-induced neurotoxicity by regulating autophagic flux. Front Pharmacol.

[229] Wagner J, Vulinovic F, Grunewald A, Unger MM, Moller JC, Klein C (2017). Acylated and unacylated ghrelin confer neuroprotection to mesencephalic neurons. Neuroscience.

[230] Zheng Y, Zhang L, Xie J, Shi L (2021). The emerging role of neuropeptides in Parkinson's disease. Front Aging Neurosci.

[231] Liu Y, Wang W, Song N, Jiao L, Jia F, Du X (2022). Ghrelin bridges DMV neuropathology and GI dysfunction in the early stages of Parkinson's disease. Adv Sci (Weinh).

[232] Suda Y, Kuzumaki N, Sone T, Narita M, Tanaka K, Hamada Y (2018). Down-regulation of ghrelin receptors on dopaminergic neurons in the substantia nigra contributes to Parkinson's disease-like motor dysfunction. Mol Brain.

[233] Song N, Wang W, Jia F, Du X, Xie A, He Q (2017). Assessments of plasma ghrelin levels in the early stages of parkinson's disease. Mov Disord.

[234] Queipo-Ortuno MI, Seoane LM, Murri M, Pardo M, Gomez-Zumaquero JM, Cardona F (2013). Gut microbiota composition in male rat models under different nutritional status and physical activity and its association with serum leptin and ghrelin levels. PLoS ONE.

[235] Wang H, Dou S, Zhu J, Shao Z, Wang C, Cheng B (2020). Ghrelin protects dopaminergic neurons against MPTP neurotoxicity through promoting autophagy and inhibiting endoplasmic reticulum mediated apoptosis. Brain Res.

[236] Morgan AH, Rees DJ, Andrews ZB, Davies JS (2018). Ghrelin mediated neuroprotection—a possible therapy for Parkinson's disease?. Neuropharmacology.

[237] Wang L, Murphy NP, Stengel A, Goebel-Stengel M, St Pierre DH, Maidment NT (2012). Ghrelin prevents levodopa-induced inhibition of gastric emptying and increases circulating levodopa in fasted rats. Neurogastroenterol Motil.

[238] Karasawa H, Pietra C, Giuliano C, Garcia-Rubio S, Xu X, Yakabi S (2014). New ghrelin agonist, HM01 alleviates constipation and L-dopa-delayed gastric emptying in 6-hydroxydopamine rat model of Parkinson's disease. Neurogastroenterol Motil.

[239] Gong B, Jiao L, Du X, Li Y, Bi M, Jiao Q (2020). Ghrelin promotes midbrain neural stem cells differentiation to dopaminergic neurons through Wnt/beta-catenin pathway. J Cell Physiol.

[240] Maruvada P, Leone V, Kaplan LM, Chang EB (2017). The human microbiome and obesity: moving beyond associations. Cell Host Microbe.

[241] Goya ME, Xue F, Sampedro-Torres-Quevedo C, Arnaouteli S, Riquelme-Dominguez L, Romanowski A (2020). Probiotic Bacillus subtilis protects against alpha-Synuclein Aggregation in C. elegans. Cell Rep.

[242] Bhattarai Y, Si J, Pu M, Ross OA, McLean PJ, Till L (2021). Role of gut microbiota in regulating gastrointestinal dysfunction and motor symptoms in a mouse model of Parkinson's disease. Gut Microbes.

[243] Sun J, Li H, Jin Y, Yu J, Mao S, Su KP (2021). Probiotic Clostridium butyricum ameliorated motor deficits in a mouse model of Parkinson's disease via gut microbiota-GLP-1 pathway. Brain Behav Immun.

[244] Lee YZ, Cheng SH, Chang MY, Lin YF, Wu CC, Tsai YC. Neuroprotective effects of Lactobacillus plantarum PS128 in a mouse model of Parkinson's disease: the role of gut microbiota and microRNAs. Int J Mol Sci. 2023;24(7). 10.3390/ijms24076794 PMC10095543 37047769

[245] Koutzoumis DN, Vergara M, Pino J, Buddendorff J, Khoshbouei H, Mandel RJ (2020). Alterations of the gut microbiota with antibiotics protects dopamine neuron loss and improve motor deficits in a pharmacological rodent model of Parkinson's disease. Exp Neurol.

[246] Zhong Z, Chen W, Gao H, Che N, Xu M, Yang L (2021). Fecal microbiota transplantation exerts a protective role in MPTP-induced Parkinson's disease via the TLR4/PI3K/AKT/NF-kappaB pathway stimulated by alpha-Synuclein. Neurochem Res.

[247] Xie Z, Zhang M, Luo Y, Jin D, Guo X, Yang W (2023). Healthy human fecal microbiota transplantation into mice attenuates MPTP-induced neurotoxicity via AMPK/SOD2 pathway. Aging Dis.

[248] Chia SJ, Tan EK, Chao YX. Historical perspective: models of Parkinson's disease. Int J Mol Sci. 2020;21(7). 10.3390/ijms21072464 PMC7177377 32252301

[249] Yin P, Li S, Li XJ, Yang W (2022). New pathogenic insights from large animal models of neurodegenerative diseases. Protein Cell.

[250] Puschhof J, Pleguezuelos-Manzano C, Clevers H (2021). Organoids and organs-on-chips: insights into human gut-microbe interactions. Cell Host Microbe.

[251] Walter J, Armet AM, Finlay BB, Shanahan F (2020). Establishing or exaggerating causality for the gut microbiome: lessons from human microbiota-associated rodents. Cell.

[252] Moossavi S, Arrieta MC, Sanati-Nezhad A, Bishehsari F (2022). Gut-on-chip for ecological and causal human gut microbiome research. Trends Microbiol.

[253] Maini Rekdal V, Bess EN, Bisanz JE, Turnbaugh PJ, Balskus EP. Discovery and inhibition of an interspecies gut bacterial pathway for Levodopa metabolism. Science. 2019;364(6445). 10.1126/science.aau6323 PMC7745125 31196984

[254] Camci G, Oguz S (2016). Association between Parkinson's disease and helicobacter pylori. J Clin Neurol.

[255] van Kessel SP, Frye AK, El-Gendy AO, Castejon M, Keshavarzian A, van Dijk G (2019). Gut bacterial tyrosine decarboxylases restrict levels of levodopa in the treatment of Parkinson's disease. Nat Commun.

[256] Spanogiannopoulos P, Bess EN, Carmody RN, Turnbaugh PJ (2016). The microbial pharmacists within us: a metagenomic view of xenobiotic metabolism. Nat Rev Microbiol.

[257] Clarke G, Sandhu KV, Griffin BT, Dinan TG, Cryan JF, Hyland NP (2019). Gut reactions: breaking down xenobiotic-microbiome interactions. Pharmacol Rev.

[258] Fahn S (2015). The medical treatment of Parkinson disease from James Parkinson to George Cotzias. Mov Disord.

[259] Hashim H, Azmin S, Razlan H, Yahya NW, Tan HJ, Manaf MR (2014). Eradication of Helicobacter pylori infection improves levodopa action, clinical symptoms and quality of life in patients with Parkinson's disease. PLoS ONE.

[260] Fasano A, Bove F, Gabrielli M, Petracca M, Zocco MA, Ragazzoni E (2013). The role of small intestinal bacterial overgrowth in Parkinson's disease. Mov Disord.

[261] Fu SC, Lee CH, Hsieh YC, Wu PH, Lin SH, Wang H (2022). A Pilot Study exploring the association of entacapone, gut microbiota, and the subsequent side effects in patients with Parkinson's disease. Front Cell Infect Microbiol.

[262] van Kessel SP, Bullock A, van Dijk G, El Aidy S (2022). Parkinson's disease medication alters small intestinal motility and microbiota composition in healthy rats. mSystems..

[263] Borzabadi S, Oryan S, Eidi A, Aghadavod E, Daneshvar Kakhaki R, Tamtaji OR (2018). The effects of probiotic supplementation on gene expression related to inflammation, insulin and lipid in patients with Parkinson's disease: a randomized, double-blind. PlaceboControlled Trial Arch Iran Med.

[264] Tamtaji OR, Taghizadeh M, Daneshvar Kakhaki R, Kouchaki E, Bahmani F, Borzabadi S (2019). Clinical and metabolic response to probiotic administration in people with Parkinson's disease: a randomized, double-blind, placebo-controlled trial. Clin Nutr.

[265] Tan AH, Lim SY, Chong KK, Maa AM, Hor JW, Lim JL (2021). Probiotics for constipation in Parkinson disease: a randomized placebo-controlled study. Neurology.

[266] Du Y, Li Y, Xu X, Li R, Zhang M, Cui Y (2022). Probiotics for constipation and gut microbiota in Parkinson's disease. Parkinsonism Relat Disord.

[267] Astarloa R, Mena MA, Sanchez V, de la Vega L, de Yebenes JG (1992). Clinical and pharmacokinetic effects of a diet rich in insoluble fiber on Parkinson disease. Clin Neuropharmacol.

[268] Becker A, Schmartz GP, Groger L, Grammes N, Galata V, Philippeit H (2022). Effects of resistant starch on symptoms, fecal markers, and gut microbiota in Parkinson's disease—the RESISTA-PD trial. Genomics Proteomics Bioinf.

[269] Barichella M, Pacchetti C, Bolliri C, Cassani E, Iorio L, Pusani C (2016). Probiotics and prebiotic fiber for constipation associated with Parkinson disease: an RCT. Neurology.

[270] Ibrahim A, Ali RAR, Manaf MRA, Ahmad N, Tajurruddin FW, Qin WZ (2020). Multi-strain probiotics (Hexbio) containing MCP BCMC strains improved constipation and gut motility in Parkinson's disease: a randomised controlled trial. PLoS ONE.

[271] Xue LJ, Yang XZ, Tong Q, Shen P, Ma SJ, Wu SN (2020). Fecal microbiota transplantation therapy for Parkinson's disease: a preliminary study. Medicine (Baltimore).

[272] Segal A, Zlotnik Y, Moyal-Atias K, Abuhasira R, Ifergane G (2021). Fecal microbiota transplant as a potential treatment for Parkinson's disease—a case series. Clin Neurol Neurosurg.

[273] Kuai XY, Yao XH, Xu LJ, Zhou YQ, Zhang LP, Liu Y (2021). Evaluation of fecal microbiota transplantation in Parkinson's disease patients with constipation. Microb Cell Fact.

[274] DuPont HL, Suescun J, Jiang ZD, Brown EL, Essigmann HT, Alexander AS (2023). Fecal microbiota transplantation in Parkinson's disease-A randomized repeat-dose, placebo-controlled clinical pilot study. Front Neurol.

[275] Hill C, Guarner F, Reid G, Gibson GR, Merenstein DJ, Pot B (2014). Expert consensus document. The International Scientific Association for Probiotics and Prebiotics consensus statement on the scope and appropriate use of the term probiotic. Nat Rev Gastroenterol Hepatol.

[276] Sanders ME, Merenstein DJ, Reid G, Gibson GR, Rastall RA (2019). Probiotics and prebiotics in intestinal health and disease: from biology to the clinic. Nat Rev Gastroenterol Hepatol.

[277] Ait-Belgnaoui A, Durand H, Cartier C, Chaumaz G, Eutamene H, Ferrier L (2012). Prevention of gut leakiness by a probiotic treatment leads to attenuated HPA response to an acute psychological stress in rats. Psychoneuroendocrinology.

[278] Corridoni D, Pastorelli L, Mattioli B, Locovei S, Ishikawa D, Arseneau KO (2012). Probiotic bacteria regulate intestinal epithelial permeability in experimental ileitis by a TNF-dependent mechanism. PLoS ONE.

[279] Musa NH, Mani V, Lim SM, Vidyadaran S, Abdul Majeed AB, Ramasamy K (2017). Lactobacilli-fermented cow's milk attenuated lipopolysaccharide-induced neuroinflammation and memory impairment in vitro and in vivo. J Dairy Res.

[280] Liao JF, Cheng YF, You ST, Kuo WC, Huang CW, Chiou JJ (2020). Lactobacillus plantarum PS128 alleviates neurodegenerative progression in 1-methyl-4-phenyl-1,2,3,6-tetrahydropyridine-induced mouse models of Parkinson's disease. Brain Behav Immun.

[281] Fang X, Tian P, Zhao X, Jiang C, Chen T (2019). Neuroprotective effects of an engineered commensal bacterium in the 1-methyl-4-phenyl-1, 2, 3, 6-tetrahydropyridine Parkinson disease mouse model via producing glucagon-like peptide-1. J Neurochem.

[282] Castelli V, d'Angelo M, Lombardi F, Alfonsetti M, Antonosante A, Catanesi M (2020). Effects of the probiotic formulation SLAB51 in in vitro and in vivo Parkinson's disease models. Aging (Albany N Y).

[283] Hsieh TH, Kuo CW, Hsieh KH, Shieh MJ, Peng CW, Chen YC, et al. Probiotics alleviate the progressive deterioration of motor functions in a mouse model of Parkinson's disease. Brain Sci. 2020;10(4). 10.3390/brainsci10040206 PMC7226147 32244769

[284] Srivastav S, Neupane S, Bhurtel S, Katila N, Maharjan S, Choi H (2019). Probiotics mixture increases butyrate, and subsequently rescues the nigral dopaminergic neurons from MPTP and rotenone-induced neurotoxicity. J Nutr Biochem.

[285] Magistrelli L, Amoruso A, Mogna L, Graziano T, Cantello R, Pane M (2019). Probiotics may have beneficial effects in Parkinson's disease: in vitro evidence. Front Immunol.

[286] Gibson GR, Hutkins R, Sanders ME, Prescott SL, Reimer RA, Salminen SJ (2017). Expert consensus document: the International Scientific Association for Probiotics and Prebiotics (ISAPP) consensus statement on the definition and scope of prebiotics. Nat Rev Gastroenterol Hepatol.

[287] Pandey KR, Naik SR, Vakil BV (2015). Probiotics, prebiotics and synbiotics—a review. J Food Sci Technol.

[288] Hutkins RW, Krumbeck JA, Bindels LB, Cani PD, Fahey G, Goh YJ (2016). Prebiotics: why definitions matter. Curr Opin Biotechnol.

[289] Markowiak P, Slizewska K. Effects of probiotics, prebiotics, and synbiotics on human health. Nutrients. 2017;9(9). 10.3390/nu9091021 PMC5622781 28914794

[290] Cencic A, Chingwaru W (2010). The role of functional foods, nutraceuticals, and food supplements in intestinal health. Nutrients.

[291] Alfonsetti M, Castelli V, d'Angelo M. Are we what we eat? Impact of diet on the gut-brain axis in Parkinson's disease. Nutrients. 2022;14(2). 10.3390/nu14020380 PMC8780419 35057561

[292] Perez-Pardo P, Kliest T, Dodiya HB, Broersen LM, Garssen J, Keshavarzian A (2017). The gut-brain axis in Parkinson's disease: possibilities for food-based therapies. Eur J Pharmacol.

[293] Dong XL, Wang X, Liu F, Liu X, Du ZR, Li RW (2020). Polymannuronic acid prevents dopaminergic neuronal loss via brain-gut-microbiota axis in Parkinson's disease model. Int J Biol Macromol.

[294] Swanson KS, Gibson GR, Hutkins R, Reimer RA, Reid G, Verbeke K (2020). The International Scientific Association for Probiotics and Prebiotics (ISAPP) consensus statement on the definition and scope of synbiotics. Nat Rev Gastroenterol Hepatol.

[295] Liu X, Du ZR, Wang X, Sun XR, Zhao Q, Zhao F (2022). Polymannuronic acid prebiotic plus Lacticaseibacillus rhamnosus GG probiotic as a novel synbiotic promoted their separate neuroprotection against Parkinson's disease. Food Res Int.

[296] Zhang F, Cui B, He X, Nie Y, Wu K, Fan D (2018). Microbiota transplantation: concept, methodology and strategy for its modernization. Protein Cell.

[297] Carlucci C, Petrof EO, Allen-Vercoe E (2016). Fecal microbiota-based therapeutics for recurrent clostridium difficile infection. Ulcerative Colitis and Obesity EBioMedicine.

[298] Hazan S (2020). Rapid improvement in Alzheimer's disease symptoms following fecal microbiota transplantation: a case report. J Int Med Res.

[299] Kang DW, Adams JB, Coleman DM, Pollard EL, Maldonado J, McDonough-Means S (2019). Long-term benefit of microbiota transfer therapy on autism symptoms and gut microbiota. Sci Rep.

[300] Li K, Wei S, Hu L, Yin X, Mai Y, Jiang C (2020). Protection of fecal microbiota transplantation in a mouse model of multiple sclerosis. Mediators Inflamm.

[301] Zhang T, Wang T, Chen X, Zhao Z, Chen Z (2022). Gut microbiota relieves inflammation in the substantia nigra of chronic Parkinson's disease by protecting the function of dopamine neurons. Exp Ther Med.

[302] Van Laar T, Boertien JM, Herranz AH (2019). Faecal transplantation, pro- and prebiotics in Parkinson's disease; hope or hype?. J Parkinsons Dis.

[303] Feng Q, Chen WD, Wang YD (2018). Gut microbiota: an integral moderator in health and disease. Front Microbiol.

[304] Kellermayer R (2019). Fecal microbiota transplantation: great potential with many challenges. Transl Gastroenterol Hepatol.

[305] Costello SP, Hughes PA, Waters O, Bryant RV, Vincent AD, Blatchford P (2019). Effect of fecal microbiota transplantation on 8-week remission in patients with ulcerative colitis: a randomized clinical trial. JAMA.

[306] Huang H, Xu H, Luo Q, He J, Li M, Chen H (2019). Fecal microbiota transplantation to treat Parkinson's disease with constipation: a case report. Medicine (Baltimore).

[307] Aizpurua O, Blijleven K, Trivedi U, Gilbert MTP, Alberdi A (2023). Unravelling animal-microbiota evolution on a chip. Trends Microbiol.

[308] Beaurivage C, Kanapeckaite A, Loomans C, Erdmann KS, Stallen J, Janssen RAJ (2020). Development of a human primary gut-on-a-chip to model inflammatory processes. Sci Rep.

[309] Jalili-Firoozinezhad S, Miranda CC, Cabral JMS (2021). Modeling the human body on microfluidic chips. Trends Biotechnol.

