# Supplementary material for: Parkinson’s disease and gut microbiota: from clinical to mechanistic and therapeutic studies
Source: Transl Neurodegener. 2023 Dec 15;12:59. doi: 10.1186/s40035-023-00392-8 (PMC10722742; doi:10.1186/s40035-023-00392-8)
Supplement: Supplementary file 1 — Additional file 1: Table S1. Microbiome alterations in clinical cohorts of Parkinson’s disease. [file 40035_2023_392_MOESM1_ESM.docx]

**Table S1** Microbiome Alterations in Clinical Cohorts of Parkinson’s disease.

| **Reference** | **Country** | **Sample**  **size** | **Control factors** | **Sample collection** | **Method** | **Microbiota alterations** | **Alpha diversity** | **Beta diversity** |
| --- | --- | --- | --- | --- | --- | --- | --- | --- |
| Scheperjans *et al*.[1] | Finland | PD: 72 non-PD: 72 | Onset age > 50 yrs; age- and sex-matched; no endocrine diseases | DNA stabilizer  stored at-80°C | 16S rRNA V1-3 | family increased: Lactobacillaceae, Verrucomicrobiaceae, Bradyrhizobiaceae, Clostridiales Incertae Sedis IV, Ruminococcaceae family decreased: Prevotellaceae | nd | sd |
| Hasegawa *et al*.[2] | Japan | PD: 52 healthy: 36 | Spouses as control | RNA stabilizer stored at 4°C | qRT-PCR | genus increased: *Lactobacillus* species decreased: *Clostridium coccoides, Bacteroides fragilis* | na | na |
| Keshavarzian et al.[3] | United States | PD: 38 healthy: 34 | Including 12 drug naïve PD | Stored at -80°C | 16S rRNA V4 | phylum increased: Bacteroidetes, Proteobacteria, Verrucomicrobia phylum decreased: Firmicutes genus increased: *Akkermansia, Oscillospira, Bacteroides* genus decreased: *Blautia, Coprococcus, Roseburia* | nd | sd |
| Unger *et al*.[4] | Germany | PD: 34 healthy: 34 | Age-matched; no special dietary habits | Stored at -35°C | qPCR | phylum decreased: Bacteroidetes family increased: Enterobacteriaceae  family decreased: Prevotellaceae, Lactobacillaceae, Enterococcaceae  genus increased: *Bifidobacterium* species decreased: *Faecalibacterium prausnitzii* | na | na |
| Bedarf  *et al*.[5] | Germany | PD: 31  non-PD: 28 | Early, L-DOPA-naïve PD; only male; age-matched; considered diet, smoking habits | Not reported | shotgun metagenomic sequencing | phylum increased: Firmicutes family/genus increased: Verrucomicrobiaceae (genus *Akkermansia*) family/genus decreased: Prevotellaceae (genus *Prevotella*), Erysipelotrichaceae (genus *Eubacterium*) species increased: *Akkermansia muciniphila, Alistipes shahii*  species decreased: *Prevotella copri, Eubacterium bioforme, Clostridium saccharolyticum* | nd | sd |
| Hill-Burns *et al*.[6] | United States | PD: 197 non-PD: 130 | 39 potential confounders including medications, diet, gastrointestinal symptoms were collected | Shipped at ambient temperature | 16S rRNA V4 | family increased: Bifidobacteriaceae, Lactobacillaceae, Tissierellaceae, Christensenellaceae, Verrucomicrobiaceae, Porphyromonadaceae, Prevotellaceae family decreased: Lachnospiraceae, Pasteurellaceae genus increased: *Akkermansia, Lactobacillus, Bifidobacterium, Parabacteroides, Prevotella* genus decreased: *Blautia,Roseburia, Faecalibacterium, Coprococcus* | na | sd |
| Hopfner *et al*.[7] | northern Germany | PD: 29 non-PD: 29 | Age-matched | Shipped at room temperature stored at -80°C | 16S rRNA V1-2 | family increased: Lactobacillaceae, Barnesiellaceae, Enterococcaceae | nd | sd |
| Li *et al*.[8] | China | PD: 24 healthy: 14 | Age, sex, BMI matched; no diabetes, infectious diseases or special diets | Stored at -80°C | 16S rRNA V3-5 | phylum increased: Actinobacteria, Proteobacteria phylum decreased: Bacteroidetes family increased: Enterobacteriaceae, Veillonellaceae, Erysipelotrichaceae, Coriobacteriaceae, Streptococcaceae, Moraxellaceae, Enterococcaceae genus increased: *Acidaminococcus, Acinetobacter, Enterococcus, Escherichia-Shigella, Megamonas, Megasphaera, Proteus, Streptococcus* genus decreased: *Blautia, Faecalibacterium, Ruminococcus* | nd | sd |
| Petrov *et al*.[9] | Russia | PD: 89 non-PD: 66 | Age-matched; BMI matched | Not reported | 16S rRNA V3-4 | genus increased: *Christensenella, Catabacter, Lactobacillus, Oscillospira, Bifidobacterium* genus decreased: *Dorea, Bacteroides, Prevotella, Faecalibacterium* species increased: *Christensenella minuta, Catabacter hongkongensis, Lactobacillus mucosae, Ruminococcus bromii, Papillibacter cinnamivorans* species decreased: *Bacteroides massiliensis, Stoquefichus massiliensis, Bacteroides coprocola, Blautia glucerasea, Dorea longicatena, Bacteroides dorei, Bacteroides plebeus, Prevotella copri, Coprococcus eutactus, Ruminococcus callidus* | sd (<) | sd |
| Heintz-Buschart *et al*.[10] | Germany | PD: 76 RBD: 21  healthy: 78 | Comorbidities and comedication were documented | Flash-frozen on dry ice | 16S/18S rRNA V4 | phylum increased: Verrucomicrobia order increased: Verrucomicrobiales family increased: Verrucomicrobiaceae genus increased: *Akkermansia* | nd | sd |
| Lin *et al*.[11] | China | PD: 75 non-PD: 45 | Age-matched; spouses as control; assessed dietary habits | Stored at -80°C | 16S rRNA V4 | phylum decreased: Tenericutes, Euryarchaeota, Firmicutes family increased: Eubacteriaceae, Bifidobacteriaceae, Aerococcaceae, Desulfovibrionaceae family decreased: Streptococcaceae, Methylobacteriaceae, Comamonadaceae, Halmonadaceae, Hyphomonadaceae, Brucellaceae, Xanthomonadaceae, Lachnospiraceae, Actinomycetaceae, Sphingomonadaceae, Pasteurellaceae, Micrococcaceae, Intrasporangiacea, Methanobacteriacea, Idiomarinaceae, Brevibacteriaceae, Gemellaceae | nd | sd |
| Qian *et al*.[12] | China | PD: 45 healthy: 45 | Spouses as control; no serious chronic illnesses (e.g., diabetes); exclude IBS | Shipped on ice  stored at -80°C | 16S rRNA V3-4 | genus increased: *Clostridium IV, Holdemania, Clostridium XVIII, Butyricicoccus, Anaerotruncus, Aquabacterium, Sphingomonas* | sd (>) | sd |
| Aho *et al*.[13] | Finland | PD: 64 control: 64 | Age- and sex-matched; assessed dietary habits, medications | DNA stabilizer stored at -80°C | 16S rRNA V3-4 | family decreased: Prevotellaceae genus increased: *Bifidobacterium* genus decreased: *Roseburia* | nd | sd |
| Barichella  *et al*.[14] | Italy | PD: 193 PSP: 22 MSA: 22  non-PD: 113 | 39 drug naïve PD; age, BMI, region matched; spouses as control; assessed dietary habits; exclude autoimmune disease, advanced-stage PD | Stored at -20°C | 16S rRNA V3-4 | phylum increased: Verrucomicrobia, Proteobacteria family increased: Verrucomicrobiaceae, Enterobacteriaceae, Christensenellaceae, Lactobacillaceae, Coriobacteriaceae, Bifidobacteriaceae family decreased: Lachnospiraceae genus increased: *Akkermansia, Parabacteroides* genus decreased: *Roseburia* | sd (>) | sd |
| Li *et al*.[15] | China | PD: 51 healthy: 48 | Onset age > 50 yrs; age, BMI, region matched; no diabetes; considered ethnicity, smoking, alcohol | Collected at home stored at -80°C | 16S rRNA V4 | class decreased: Bacilli order decreased: Lactobacillales  family decreased: Bacteroidales_S24–7_group, Lactobacillaceae genus decreased: *Lactobacillus* | sd (>) | sd (<) |
| Li *et al*.[16] | China | PD: 10 non-PD: 10 | Age > 65 yrs; considered alcohol and medications | DNA/RNA stabilizer stored at -80°C | 16S rRNA V3-4 | phylum increased: Hydrogenoanaerobacterium class increased: Verrucomicrobiae order decreased: Bacteroidales family increased: Ruminococcaceae, Porphyromonadaceae, Pasteurellaceae, Lachnospiraceae_NK4A  family decreased: Leuconostocaceae, Clostridieceae genus increased: *Akkermansia* | na | sd |
| Pietrucci *et al*.[17] | Italy | PD: 80 healthy: 72 | Considered dietary/life habits | DNA stabilizer | 16S rRNA V3-4 | family increased: Lactobacillaceae, Enterobacteriaceae, Enterococcaceae family decreased: Lachnospiraceae | nd | sd |
| Weis *et al*.[18] | Germany | PD: 34 healthy: 25 | Age-Matched; no special dietary habits or restrictions | Stored at -20°C | 16S rRNA V4-5 | genus increased: *Peptoniphilus* genus decreased: *Faecalibacterium, Fusicatenibacter* | Chao 1 index: sd (<)  Shannon and Simpson index: nd | nd |
| Jin *et al*.[19] | China | PD: 72 (59 OPD, 13 NPD) healthy: 68 | Age- and lifestyle-matched; spouses or family members as control; exclude unstable medical, neurological, or psychiatric illness; | DNA stabilizer  stored at -80°C | 16S rRNA V3-4 | OPD vs. HCs: genus increased: *Bifidobacterium, Alistipes, Klebsiella, Sellimonas, Catenisphaera, Tyzzerella* genus decreased: *Lactobacillus, Streptococcus, Enterobacter, Citrobacter, Hungatella, Erysipelatoclostridium, Veillonella* NPD vs. HCs: family decreased: Streptococcaceae genus decreased: *Streptococcus, Paraprevotella* | nd | NPD vs. HC: weighted UniFrac: sd unweighted UniFrac: nd OPD vs. HC: weighted UniFrac: nd unweighted UniFrac: sd |
| Cirstea *et al*.[20] | Canada | PD: 197 healthy: 103 | Aged 40–85 yrs, onset age 40–80 yrs, disease duration ≤ 12 yrs; age-matched; collected  medications, diet, and demographics | DNA stabilizer stored at -80°C | 16S rRNA V4 | family increased: Christensenellaceae, Desulfovibrionaceae family decreased: Lachnospiraceae genus increased: *Bilophila, Akkermansia* genus decreased: *Roseburia, Faecalibacterium* | nd | sd |
| Cosma-Grigorov *et al*.[21] | Germany | PD: 71 non-PD: 30 | Exclude H&Y stage 5 PD; spouses or relatives as control; assessed nutritional factors (e.g. coffee consumption) and physical activity | DNA stabilizer stored at -80°C | 16S rRNA V3-4 | phylum decreased: Firmicutes class increased: Betaproteaobacteria order increased: Burkholderiales family increased: Sutterellaceae family decreased: Lachnospiraceae genus decreased: *Faecalibacterium, Fusicatenibacter, Gemmiger,* *Lachnospiracea incertae sedis* | Shannon and Chao1 index: nd Simpson index: sd (<) | sd |
| Hegelmaier *et al*.[22] | Germany | PD: 54 non-PD: 32 | Sex-matched; BMI matched; considered meat consumption frequency; exclude serious concomitant disease | Snap frozen at -80°C | 16S rRNA V1-3 | phylum increased (trending): Actinobacteria, Firmicutes, Proteobacteria phylum decreased (trending): Bacteroidetes class increased (trending): Negativicutes family decreased (trending): Prevotellaceae genus decreased (trending): *Butyricimonas, Odoribacter* | na | nd |
| Nishiwaki *et al*.[23] | Japan | PD: 223 healthy: 137 | Spouses as control; no chronic illnesses(e.g., diabetes, heart failure) | Shipped at 0°C | 16S rRNA V3-4 | family increased: Akkermansiaceae family decreased: Lachnospiraceae ND3007 genus increased: *Akkermansia, Catabacter* genus decreased: *Roseburia, Faecalibacterium* | sd | sd |
| Qian *et al*.[24] | China | training set PD: 40 healthy: 40 validation set PD: 78 healthy: 75 MSA: 40 AD: 25 | Spouses as partial control; no serious illness (e.g. heart failure); no chronic  disease (e.g. diabetes); considered lifestyle factors, medications | Shipped on ice  stored at -80°C | shotgun metagenomic sequencing | kingdom increased: Archaea, Viruses phylum increased: Synergistetes, Verrucomicrobia, Viruses_noname class increased: Bacilli, Deltaproteobacteria, Synergistia, Verrucomicrobiae, Viruses_noname order increased: Lactobacillales  family increased: Carnobacteriaceae, Lactobacillaceae, Rikenellaceae, Streptococcaceae, Synergistaceae genus increased: *Alistipes, Enterobacter, Gordonibacter, Granulicatella, Holdemania, Lactobacillus, Streptococcus* species increased: *Clostridium_asparagiforme, Clostridium_leptum, Enterobacter_cloacae, Gordonibacter_pamelaeae, Granulicatella_unclassified, Holdemania_filiformis, Lachnospiraceae_bacterium 1_1_57FAA, Lachnospiraceae_bacterium 3_1_57FAA_CT1, Lactobacillus_salivarius, Paraprevotella_clara, Streptococcus_anginosus, Streptococcus_salivarius, Streptococcus_thermophilus* | sd (>) | sd |
| Ren *et al*.[25] | China | PD-MCI: 13 PD-NC: 14 healthy: 13 | Spouses as control; age-matched; BMI matched; no serious  chronic illnesses (e.g., hyperlipidemia, diabetes); assessed dietary habits | Stored at -80°C | 16S rRNA V3-4 | PD-NC vs. PD-MCI and HC: genus increased: *Blautia,Ruminococcus* PD- MCI vs. PD-NC and HC: family increased: Rikenellaceae, Ruminococcaceae genus increased: *Alistipes, Barnesiella, Butyricmonas, Odoribacter, Anaerotruncus* PD-NC and PD-MCI vs. HC: genus decreased: *Veillonella* | sd (>) | HC vs. PD-NC: sd HC vs. PD-MCI: nd |
| Vascellari *et al*.[26] | Italy | PD: 64 healthy: 51 | Spouses or family members as control; exclude internal  medicine, neurological, or unstable psychiatric illness | Not reported | 16S rRNA V3-4 | family increased: Veillonellaceae family decreased: Lachnospiraceae, Enterobacteriaceae, Brevibacteriaceae, Aphanizomenonaceae  genus increased: *Veillonella* genus decreased: *Blautia, Butyrivibrio, Coprococcus, Candidatus Blochmannia, Brevibacterium, Dolichospermum* | nd | sd |
| Vidal-Martinez *et al*.[27] | United States | PD: 9 healthy: 13 | No smoking or recreational drug usage; exclude dementia or cancer | DNA stabilizer  stored at -80°C | 16S rRNA V3-4 | genus increased: *Akkermansia* | nd | nd |
| Wallen *et al*.[28] | United States | dataset 1 PD: 212 non-PD: 136 dataset 2 PD: 323 non-PD: 184 | Considered diet, medications, environmental and family history | Shipped at ambient temperature | 16S rRNA V4 | genus increased: *Porphyromonas, Prevotella, Corynebacterium_1, Bifidobacterium, Lactobacillus*  genus decreased: *Faecalibacterium, Agathobacter, Blautia, Roseburia, Butyricicoccus, Fusicatenibacter, Lachnospira, Lachnospiraceae_ND3007, Lachnospiraceae_UCG-004, Oscillospira* | na | sd |
| Baldini *et al*.[29] | Luxembourg | PD: 147 healthy: 162 | Age ≥ 50 yrs; exclude cancer and pregnancy | DNA stabilizer shipped at room temperature | 16S rRNA V3-4 | genus increased: *Christensenella, Anaerotruncus, Lactobacillus, Turicibacter, Streptococcus, Akkermansia, Bilophila, Acidaminococcus* genus decreased: *Lactobacillus*  species increased: *Christensenella minuta, Anaerotruncus colihominis, Akkermansia muciniphila, Ruminococcus bromii, Ruminococcus torques* species decreased: *Turicibacter sanguinis, Roseburia intestinalis* | Shannon index: nd  species richness: sd (>) | sd |
| Zhang *et al*.[30] | China | PD: 63 Control: 137 | 63 spouses as partial control; no diabetes and psychiatric illness | Stored at -80°C | 16S rRNA V4 | phylum increased: Firmicutes, Actinobacteria,Verrucomicrobia phylum decreased: Bacteroidetes, Fusobacteria genus increased: *Oscillospira, Akkermansia* genus decreased: *Fusobacterium* | sd (>) | sd |
| Rosario *et al*.[31] | Germany | PD: 26  Control: 25 | Early, L-DOPA-naïve PD; only male; 11 healthy controls, 14 diseased controls had cardiovascular risk factors | Not reported | shotgun metagenomic sequencing | order increased: Candidatus gastranaerophilales species increased: *Akkermansia muciniphila, Alistipes shahii, Alistipes obesi, Alistipes ihumii* species decreased: *Prevotella copri, Clostridium saccharolyticum, Desulfibrio piger* | na | na |
| Tan *et al*.[32] | Malaysia | PD: 104 non-PD: 96 | 91 spouse, 5 sibling as control; considered diet, lifestyle and housing condition | Immediately put on ice stored at -20°C | 16S rRNA V3-4 | class increased: Gammaproteobacteria family increased: Christensenellaceae, Verrucomicrobiaceae, Synergistaceae, Catabacteriaceae, Lactobacillaceae genus increased: *Cloacibacillus, Catabacter, Christensenella, Butyrivibrio, Bifidobacterium, Megasphaera* species increased: *Bacteroides fragilis, Lactobacillus acidophilus* | nd | na |
| Lubomski *et al*.[33] | Australia | PD: 21 healthy: 10 | PD initiating DBS or LCIG; spouses as control; considered diet, lifestyle and comorbidities | Stored at -80°C | 16S rRNA V3-4 | phylum increased: Verrucomicrobia  phylum decreased: Firmicutes, Bacteroidetes order increased: Verrucomicrobiales order decreased: Pasteurellales family increased: Verrucomicrobiaceae  family decreased: Pasteurellaceae, Veillonellaceae genus increased: *Akkermansia*  genus decreased: *Blautia, Faecalibacterium, Roseburia, Fusicatenibacter, Haemophilus, Gemmiger, Butyricicoccus, Streptococcus* | nd | sd |
| Wallen *et al*.[34] | United States | PD: 490 neurologically healthy: 234 | Region-matched; 55% controls were spouses | Collected at home and stored at -20°C | shotgun metagenomic sequencing | genus: 23 ↑, 11 ↓  species: 55 ↑, 29 ↓ | na | sd |
| Zhang *et al*.[35] | United States | PD: 96  non-PD: 74 | Newly diagnosed PD; considered environmental factors; no immunocompromised | Collected at home and stored at -80°C | 16S rRNA V4 | phylum increased: Proteobacteria, Verrucomicrobiota, Actinobacteria  genus increased: *Akkermansia, Enterococcus, Hungatella,* | sd (<) | sd |

Note: <, ↓: a lower abundance in PD compared to Control; >, ↑: a higher abundance in PD compared to Control.

PD: Parkinson’s disease, nd: no difference, sd: significant difference, na: not available, L-DOPA: Levodopa, BMI: body mass index, RBD: rapid eye movement sleep behavior disorder, IBS: irritable bowel syndrome, PSP: progressive supranuclear palsy, MSA: multiple system atrophy, OPD: PD for ≥ 1 year, NPD: new PD, AD: Alzheimer's disease; MCI: mild cognitive impairment; NC: normal cognition, DBS: deep brain stimulation, LCIG: levodopa–carbidopa intestinal gel.

# References

1. Scheperjans F, Aho V, Pereira PA, Koskinen K, Paulin L, Pekkonen E, et al. Gut microbiota are related to Parkinson's disease and clinical phenotype. Mov Disord. 2015;30(3):350-8.

2. Hasegawa S, Goto S, Tsuji H, Okuno T, Asahara T, Nomoto K, et al. Intestinal Dysbiosis and Lowered Serum Lipopolysaccharide-Binding Protein in Parkinson's Disease. PLoS One. 2015;10(11):e0142164.

3. Keshavarzian A, Green SJ, Engen PA, Voigt RM, Naqib A, Forsyth CB, et al. Colonic bacterial composition in Parkinson's disease. Mov Disord. 2015;30(10):1351-60.

4. Unger MM, Spiegel J, Dillmann KU, Grundmann D, Philippeit H, Burmann J, et al. Short chain fatty acids and gut microbiota differ between patients with Parkinson's disease and age-matched controls. Parkinsonism Relat Disord. 2016;32:66-72.

5. Bedarf JR, Hildebrand F, Coelho LP, Sunagawa S, Bahram M, Goeser F, et al. Functional implications of microbial and viral gut metagenome changes in early stage L-DOPA-naive Parkinson's disease patients. Genome Med. 2017;9(1):39.

6. Hill-Burns EM, Debelius JW, Morton JT, Wissemann WT, Lewis MR, Wallen ZD, et al. Parkinson's disease and Parkinson's disease medications have distinct signatures of the gut microbiome. Mov Disord. 2017;32(5):739-49.

7. Hopfner F, Kunstner A, Muller SH, Kunzel S, Zeuner KE, Margraf NG, et al. Gut microbiota in Parkinson disease in a northern German cohort. Brain Res. 2017;1667:41-45.

8. Li W, Wu X, Hu X, Wang T, Liang S, Duan Y, et al. Structural changes of gut microbiota in Parkinson's disease and its correlation with clinical features. Sci China Life Sci. 2017;60(11):1223-33.

9. Petrov VA, Saltykova IV, Zhukova IA, Alifirova VM, Zhukova NG, Dorofeeva YB, et al. Analysis of Gut Microbiota in Patients with Parkinson's Disease. Bull Exp Biol Med. 2017;162(6):734-37.

10. Heintz-Buschart A, Pandey U, Wicke T, Sixel-Doring F, Janzen A, Sittig-Wiegand E, et al. The nasal and gut microbiome in Parkinson's disease and idiopathic rapid eye movement sleep behavior disorder. Mov Disord. 2018;33(1):88-98.

11. Lin A, Zheng W, He Y, Tang W, Wei X, He R, et al. Gut microbiota in patients with Parkinson's disease in southern China. Parkinsonism Relat Disord. 2018;53:82-88.

12. Qian Y, Yang X, Xu S, Wu C, Song Y, Qin N, et al. Alteration of the fecal microbiota in Chinese patients with Parkinson's disease. Brain Behav Immun. 2018;70:194-202.

13. Aho VTE, Pereira PAB, Voutilainen S, Paulin L, Pekkonen E, Auvinen P, et al. Gut microbiota in Parkinson's disease: Temporal stability and relations to disease progression. EBioMedicine. 2019;44:691-707.

14. Barichella M, Severgnini M, Cilia R, Cassani E, Bolliri C, Caronni S, et al. Unraveling gut microbiota in Parkinson's disease and atypical parkinsonism. Mov Disord. 2019;34(3):396-405.

15. Li C, Cui L, Yang Y, Miao J, Zhao X, Zhang J, et al. Gut Microbiota Differs Between Parkinson's Disease Patients and Healthy Controls in Northeast China. Front Mol Neurosci. 2019;12:171.

16. Li F, Wang P, Chen Z, Sui X, Xie X, Zhang J. Alteration of the fecal microbiota in North-Eastern Han Chinese population with sporadic Parkinson's disease. Neurosci Lett. 2019;707:134297.

17. Pietrucci D, Cerroni R, Unida V, Farcomeni A, Pierantozzi M, Mercuri NB, et al. Dysbiosis of gut microbiota in a selected population of Parkinson's patients. Parkinsonism Relat Disord. 2019;65:124-30.

18. Weis S, Schwiertz A, Unger MM, Becker A, Fassbender K, Ratering S, et al. Effect of Parkinson's disease and related medications on the composition of the fecal bacterial microbiota. NPJ Parkinsons Dis. 2019;5:28.

19. Jin M, Li J, Liu F, Lyu N, Wang K, Wang L, et al. Analysis of the Gut Microflora in Patients With Parkinson's Disease. Front Neurosci. 2019;13:1184.

20. Cirstea MS, Yu AC, Golz E, Sundvick K, Kliger D, Radisavljevic N, et al. Microbiota Composition and Metabolism Are Associated With Gut Function in Parkinson's Disease. Mov Disord. 2020;35(7):1208-17.

21. Cosma-Grigorov A, Meixner H, Mrochen A, Wirtz S, Winkler J, Marxreiter F. Changes in Gastrointestinal Microbiome Composition in PD: A Pivotal Role of Covariates. Front Neurol. 2020;11:1041.

22. Hegelmaier T, Lebbing M, Duscha A, Tomaske L, Tonges L, Holm JB, et al. Interventional Influence of the Intestinal Microbiome Through Dietary Intervention and Bowel Cleansing Might Improve Motor Symptoms in Parkinson's Disease. Cells. 2020;9(2).

23. Nishiwaki H, Ito M, Ishida T, Hamaguchi T, Maeda T, Kashihara K, et al. Meta-Analysis of Gut Dysbiosis in Parkinson's Disease. Mov Disord. 2020;35(9):1626-35.

24. Qian Y, Yang X, Xu S, Huang P, Li B, Du J, et al. Gut metagenomics-derived genes as potential biomarkers of Parkinson's disease. Brain. 2020;143(8):2474-89.

25. Ren T, Gao Y, Qiu Y, Jiang S, Zhang Q, Zhang J, et al. Gut Microbiota Altered in Mild Cognitive Impairment Compared With Normal Cognition in Sporadic Parkinson's Disease. Front Neurol. 2020;11:137.

26. Vascellari S, Palmas V, Melis M, Pisanu S, Cusano R, Uva P, et al. Gut Microbiota and Metabolome Alterations Associated with Parkinson's Disease. mSystems. 2020;5(5).

27. Vidal-Martinez G, Chin B, Camarillo C, Herrera GV, Yang B, Sarosiek I, et al. A Pilot Microbiota Study in Parkinson's Disease Patients versus Control Subjects, and Effects of FTY720 and FTY720-Mitoxy Therapies in Parkinsonian and Multiple System Atrophy Mouse Models. J Parkinsons Dis. 2020;10(1):185-92.

28. Wallen ZD, Appah M, Dean MN, Sesler CL, Factor SA, Molho E, et al. Characterizing dysbiosis of gut microbiome in PD: evidence for overabundance of opportunistic pathogens. NPJ Parkinsons Dis. 2020;6:11.

29. Baldini F, Hertel J, Sandt E, Thinnes CC, Neuberger-Castillo L, Pavelka L, et al. Parkinson's disease-associated alterations of the gut microbiome predict disease-relevant changes in metabolic functions. BMC Biol. 2020;18(1):62.

30. Zhang F, Yue L, Fang X, Wang G, Li C, Sun X, et al. Altered gut microbiota in Parkinson's disease patients/healthy spouses and its association with clinical features. Parkinsonism Relat Disord. 2020;81:84-88.

31. Rosario D, Bidkhori G, Lee S, Bedarf J, Hildebrand F, Le Chatelier E, et al. Systematic analysis of gut microbiome reveals the role of bacterial folate and homocysteine metabolism in Parkinson's disease. Cell Rep. 2021;34(9):108807.

32. Tan AH, Chong CW, Lim SY, Yap IKS, Teh CSJ, Loke MF, et al. Gut Microbial Ecosystem in Parkinson Disease: New Clinicobiological Insights from Multi-Omics. Ann Neurol. 2021;89(3):546-59.

33. Lubomski M, Xu X, Holmes AJ, Yang JYH, Sue CM, Davis RL. The impact of device-assisted therapies on the gut microbiome in Parkinson's disease. J Neurol. 2022;269(2):780-95.

34. Wallen ZD, Demirkan A, Twa G, Cohen G, Dean MN, Standaert DG, et al. Metagenomics of Parkinson's disease implicates the gut microbiome in multiple disease mechanisms. Nat Commun. 2022;13(1):6958.

35. Zhang K, Paul KC, Jacobs JP, Chou HL, Duarte Folle A, Del Rosario I, et al. Parkinson's Disease and the Gut Microbiome in Rural California. J Parkinsons Dis. 2022;12(8):2441-52.
